# SciClone: Inferring Clonal Architecture and Tracking the Spatial and Temporal Patterns of Tumor Evolution

**DOI:** 10.1371/journal.pcbi.1003665

**Published:** 2014-08-07

**Authors:** Christopher A. Miller, Brian S. White, Nathan D. Dees, Malachi Griffith, John S. Welch, Obi L. Griffith, Ravi Vij, Michael H. Tomasson, Timothy A. Graubert, Matthew J. Walter, Matthew J. Ellis, William Schierding, John F. DiPersio, Timothy J. Ley, Elaine R. Mardis, Richard K. Wilson, Li Ding

**Affiliations:** 1 The Genome Institute, Washington University, St. Louis, Missouri, United States of America; 2 Department of Internal Medicine, Division of Oncology, Washington University School of Medicine, St. Louis, Missouri, United States of America; 3 Department of Genetics, Washington University, St. Louis, Missouri, United States of America; 4 Siteman Cancer Center, Barnes-Jewish Hospital, Washington University School of Medicine, St. Louis, Missouri, United States of America; 5 Massachusetts General Hospital, Boston, Massachusetts, United States of America; 6 Liggins Institute, Auckland, New Zealand; ETH Zurich, Switzerland

## Abstract

The sensitivity of massively-parallel sequencing has confirmed that most cancers are oligoclonal, with subpopulations of neoplastic cells harboring distinct mutations. A fine resolution view of this clonal architecture provides insight into tumor heterogeneity, evolution, and treatment response, all of which may have clinical implications. Single tumor analysis already contributes to understanding these phenomena. However, cryptic subclones are frequently revealed by additional patient samples (e.g., collected at relapse or following treatment), indicating that accurately characterizing a tumor requires analyzing multiple samples from the same patient. To address this need, we present SciClone, a computational method that identifies the number and genetic composition of subclones by analyzing the variant allele frequencies of somatic mutations. We use it to detect subclones in acute myeloid leukemia and breast cancer samples that, though present at disease onset, are not evident from a single primary tumor sample. By doing so, we can track tumor evolution and identify the spatial origins of cells resisting therapy.

## Author Summary

Sequencing the genomic DNA of cancers has revealed that tumors are not homogeneous. As a tumor grows, new mutations accumulate in individual cells, and as these cells replicate, the mutations are passed on to their offspring, which comprise only a portion of the tumor when it is sampled. We present a method for identifying the fraction of cells containing specific mutations, clustering them into subclonal populations, and tracking the changes in these subclones. This allows us to follow the clonal evolution of cancers as they respond to chemotherapy or develop therapy resistance, processes which may radically alter the subclonal composition of a tumor. It also gives us insight into the spatial organization of tumors, and we show that multiple biopsies from a single breast cancer may harbor different subclones that respond differently to treatment. Finally, we show that sequencing multiple samples from a patient's tumor is often critical, as it reveals cryptic subclones that cannot be discerned from only one sample. This is the first tool that can efficiently leverage multiple samples to identify these as distinct subpopulations of cells, thus contributing to understanding the biology of the tumor and influencing clinical decisions about therapy.

## Introduction

Cancer is a disease largely driven by accumulated somatic mutations. Many of these are clonal mutations and occur in the founding cell to initiate disease. These become uniformly present in the tumor by propagating to that cell's progeny during clonal expansion. Others are subclonal events, which occur in an existing neoplastic cell and are then passed on only to the subpopulation of cells derived from it. The result of this accumulation of mutations is that tumors are composed of a heterogeneous mixture of cells. These subpopulations compete and evolve [1]–[3], and the mutations “captured” [4] in subsets of cells during this evolution serve as a genetic signature of the resulting (sub)clones.

Recently, high resolution glimpses of this clonal heterogeneity have been provided by next-generation sequencing [4]–[14], SNP array [6], [10], [13], [15], and array comparative genomic hybridization [16], [17] platforms. Single-cell sequencing [16], [18]–[20] may eventually address this heterogeneity directly without the confounding effects of mixing cell types, but technical challenges, such as allele dropout [21], remain. There are also pragmatic concerns about the large number of cells that must be sequenced to establish the heterogeneity of a given sample. The emerging picture from these studies, across a diversity of solid [6], [8], [9], [11], [13], [16] and hematological [4], [5], [7], [10], [12], [14], [15], [17], [22] disorders, is that tumors are both spatially [9], [13], [16] and temporally [4]–[17] heterogeneous and are frequently comprised of a single founding clone and several subclones.

Increasing evidence suggests that intra-tumor heterogeneity and clonal architecture have clinical implications [3], [23], [24] and contribute to therapy resistance [25]. Several studies have linked the presence of subclones to poor clinical outcome, as in chronic lymphocytic leukemia (CLL) [10], or to increased risk of progression to malignancy, as in Barrett's esophagus [26] and multiple myeloma (MM) [17]. Subclonal mutations can drive resistance as well, as shown in *EGFR*-mutant non-small cell lung cancers [27], [28]. Studies in chronic myeloid leukemia have also demonstrated that drug-resistant subclones may harbor aggressive mutations that are restrained by more fit, but indolent clones [2], [3]. In these cases, therapeutic application of imatinib leads to competitive release of BCR-ABL mutant subclones, which renders the therapy ineffective [3], [29]. Thus, designing effective second line therapies requires a deep understanding of both a cancer's underlying mutations and how it's clonal structure evolves in response to treatment.

Existing methods [6], [10], [11], [22], [30]–[32] have been useful for inferring clonal architecture and its consequences, e.g., that putative driver mutations in *SF3B1* and *TP53* in CLL [10] and in *PIK3CA* and *PTEN* in triple-negative breast cancer [11] may arise during late-emerging, subclonal diversification [6] rather than as founding lesions. Recent results suggest that accurately describing the subclonal composition and evolution of tumors requires sampling cancer cells across multiple time points or spatially-distinct regions [3], [23], [25]. Current studies of distant metastases and of spatial heterogeneity are collecting as many as six to twenty samples [9], [13], [16], [18]. The scale of these ambitious studies will challenge existing methods. For example, histogram-based approaches to representing clonal markers [10], [31] are attractive in avoiding model assumptions in low dimensions (i.e., with few samples), but with many samples will suffer from exponential computational complexity. Several approaches [6], [10], [11], [22], [30], [31] leverage Markov chain Monte Carlo (MCMC) techniques, but these, too, are computationally demanding and rely on assumptions about chain convergence.

Most existing methods [6], [10], [22], [31] inferring clonality from copy number alterations (CNAs) avoid additional computational overhead by making the simplifying assumption that the tumor sample is “monogenomic” [31] and does not harbor subclonal copy-number events. Contrary to their assumptions, these methods have detected such subclonal events in CLL [10], though without being able to correct for them. Similar subclonal events have been detected in MM (Ref. [33] and B.S.W., R.V., and M.H.T, data not shown). These methods introduce uncertainty, through the probabilistic inference of allele-specific copy numbers, and error, by ignoring subclonal CNAs. A recent approach [32] does generalize to subclonal CNAs, but also suffers from computational inefficiencies when extended beyond the simple case of clonal CNAs. A method which could avoid the uncertainty of deconvolving subclonal CNAs and operated with significantly lower computational demands would benefit studies aiming to understand the evolution of cancer.

To address these needs, we introduce SciClone, a method for estimating the number and content of subclones across one or many samples. It focuses primarily on variants in copy-number neutral, loss of heterozygosity (LOH)-free portions of the genome, which allows for the highest-confidence quantification of variant allele frequencies (VAF) and inference of clonality. As with other tools [6], [11], [22], [30], regions of CNA and LOH are provided as inputs after having been inferred from whole-exome sequencing (WES, e.g., via ASCAT [22], [34], cn.MOPS [35]–[37], or VarScan 2 [38]), whole-genome sequencing (WGS, e.g., via HMMcopy/APOLLOH [11], [39] or VarScan 2 [38]), or SNP arrays (e.g., via ASCAT [6], [22], [34]). SNVs of sufficient depth are provided by WES or first discovered by WGS and subsequently deeply sequenced in a targeted fashion. The approach is not limited to SNVs, but is amenable to any event that can be described as a frequency. In particular, we demonstrate the integration of copy number events and discuss how copy-altered VAFs could be accommodated.

Computational efficiency is achieved by clustering the VAFs using a variational Bayesian mixture model [40] (VBMM), which differs substantially from the Dirichlet process models previously used to infer subclones [6], [10], [11], [22], [30], [31]. VBMMs similarly automatically infer the number of clusters and provide a probabilistic interpretation of clustering, but their deterministic nature allows them to scale to high dimension, where they enjoy efficiency advantages [40] over stochastic MCMC techniques employed by existing clonality detection methods [6], [10], [11], [22], [30], [31]. Further, the variational Bayesian approach provides a computational termination condition more straightforward than monitoring techniques [41] required of MCMC. Though VBMMs are heuristic, and their approximations occasionally result in sub-optimal solutions [42], we demonstrate their effectiveness here through simulation and application to several real tumor data sets. In particular, SciClone advances our preliminary [14] variational Bayesian beta mixture modeling approach for clustering VAFs in a single sample by: (1) applying the standard technique of factorizing the density over samples [43], [44] to extend applicability to an arbitrary number of samples, (2) replacing our previous *ad hoc* notion of cluster overlap with a quantitative measure [45], [46], (3) leveraging the probabilistic nature of VBMMs to quantify a variant's likelihood of belonging to a cluster via a 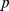-value, and (4) offering alternative binomial and Gaussian mixture models.

We demonstrate SciClone by inferring low-frequency subclones from a single MM sample and by quantitatively assessing the clonality of driver mutations in (potentially noisy) WES-derived data. We extend this approach to accommodate multiple samples and apply it to track clonal evolution of an acute myeloid leukemia (AML) tumor to relapse in response to therapy. As a further example of SciClone's scalability and utility in correlating mutations across samples, we examine spatial heterogeneity and aromatase-inhibitor resistance within three samples from a single breast cancer patient. In both the AML and breast cancer data sets, our analysis reveals subclones present in the primary tumor but not discernible from a single primary tumor sample. This reinforces the necessity of analyzing multiple patient-derived tumor samples to elucidate the full complexity of a cancer's heterogeneity.

The SciClone package is available at http://github.com/genome/sciclone.

## Results

### Mixture modeling objectively identifies subclones

**Figure 1:**
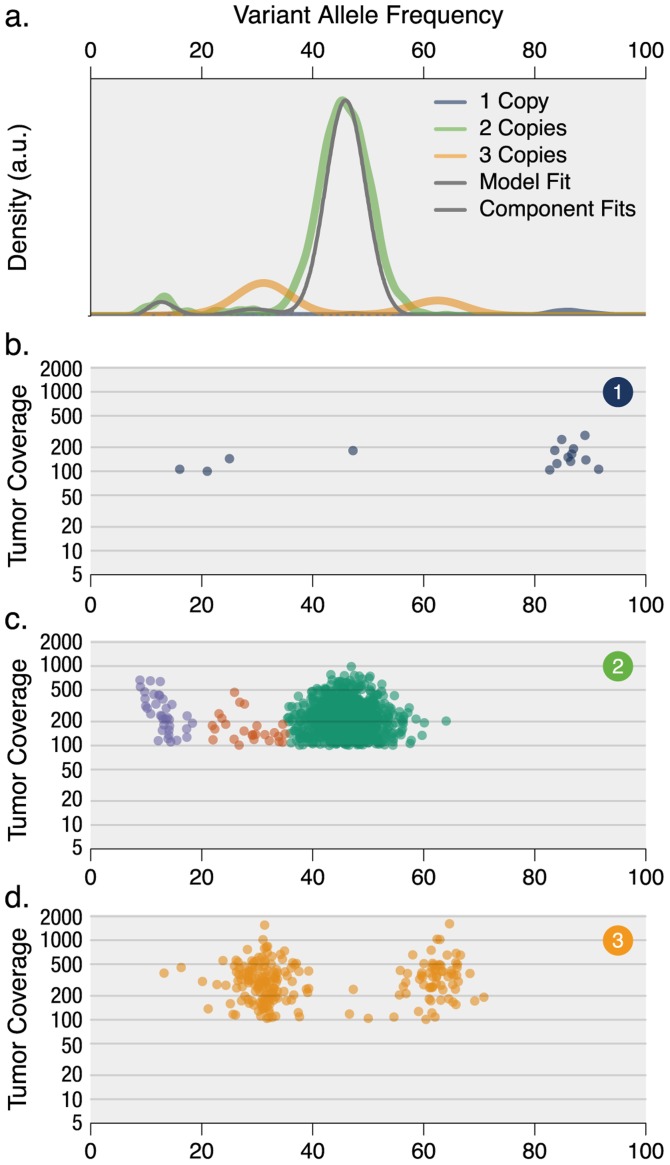
Inferring subclonal architecture objectively in multiple myeloma. (a) Kernel density plots of VAFs across regions with copy number one, two, or three, posterior predictive densities summed over all clusters for copy number neutral variants, and posterior predictive densities for each cluster/component. (b-d) VAFs plotted versus read depth for each of the three copy number regions. (c) Three mutation clusters (green, dark orange, and blue) were detected using variants from copy number neutral segments. (d) Two clusters centered at VAF 31% and 62% were detected from variants in copy number three segments; they likely result from single-copy amplification of the wild-type or the mutant allele of mutations in the founding clone.

Many tumors are highly heterogeneous and visualizations of somatic VAFs reveal high-density aggregations that correspond to specific subpopulations of cells (Fig. 1). To test our ability to detect and segregate these clusters, we used 2,018 validated, deep-sequenced (median depth 188x), genome-wide somatic SNVs from a primary MM tumor (M.H.T., et al., in preparation). These formed a high density region near 50% VAF, as expected of heterozygous SNVs in the founding clone of a nearly pure tumor sample (Figs. 1a and c). The actual average VAF of this founding clone is slightly less (46.1%), reflecting a small amount of normal cell admixture and a tumor purity 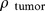 of 0.922. Lower VAFs correspond to subclone-specific mutations that arose later in the tumor's expansion; a cluster of such VAFs thus represents a subclonal population, whose cells contain all of the founding clone mutations, as well as these subclonal mutations.

This tumor is hyperdiploid with characteristic trisomies of chromosomes 3, 5, 7, 9, 11, 15, 19, and 21. Founding clone mutations in these (and other) copy number altered regions are shifted in a predictable pattern, with a doubling of VAFs in regions of single-copy deletion where only the variant allele remains (Fig. 1b). In regions of single-copy amplification, mutations group near the expected frequency of 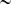
31% where the wild-type allele is amplified (i.e., ∼1 variant allele/3 total alleles) or 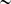
62% when the mutant allele is amplified (i.e., 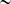
2 variant alleles/3 total alleles; Fig. 1d). A more careful calculation that incorporates the tumor purity 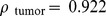 gives an expected frequency of the mutant-amplified cluster, 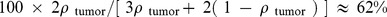, very close to the observed frequency of 63%. The same holds for the wild-type-amplified cluster.

In this patient, these wild-type amplified SNVs occur at similar VAFs to subclonal events that are copy number neutral (red circles in Fig 1c). Disambiguating the two would require inference of allele- (and subclone-) specific copy number profiles, with its attendant uncertainty. To confirm that the two cases are in fact distinct and not an artifact of inaccurate copy number calls, we verified that none of the subclonal event VAFs in putative copy-number neutral regions reside instead on trisomic chromosomes. Further, they are not restricted to one or a few chromosomes, whose amplification or deletion might not have been detected, thus leading to apparent subclonal events.

To obtain a more objective view of this sample's clonal architecture, we identified low-frequency subclones by clustering the VAFs from copy number neutral, LOH-free, non-repetitive regions of the genome using SciClone, which uses an approach based on variational Bayesian beta mixture modeling [43], [44], [47]. The method automatically infers the optimal number of clusters, based on an initial overestimation of their expected number (ten, unless specified otherwise). In this MM sample, SciClone detected three clusters (Fig. 1): the first with average VAF of 46.1%, and two lower-frequency clusters with average VAFs of 32.0% and 11.9% (Fig. 1). Each cluster is represented by a posterior predictive density (see Materials and Methods), which provides the probability of a VAF given the observed data (and subsequent model fit). These densities probabilistically define boundaries between clusters, including the visually ambiguous separation between the highest-frequency cluster and the cluster with average VAF of 32.0%.

As a comparison for our results, we applied an MCMC method, PyClone [11], [30], to the same variants in copy-number neutral, LOH-free regions. PyClone recapitulated the presence of the minor clusters, though with increased computational demands (Table S1). We tested PyClone's ability to integrate copy-number altered SNVs expected to be in the founding clone and found that it often assigned these sites to independent clusters.

As a second point of comparison, we applied THetA [32], a method that infers clonal architecture based on copy number events alone. For reasons of computational complexity, we applied it to a limited number of segments with representative copy number states. THetA detected clonal amplifications that occurred in 89.8% of the cells (44.9% VAF) and a subclonal deletion that occurred in 54.1% of the cells (27.0% VAF). We integrated these data and SciClone clustering of all CNA and SNV VAFs revealed that the THetA-inferred CNA VAFs support the presence of subclones originally inferred from SNVs alone (Fig. S1).

In some samples, ordering of subclonal VAFs may reveal the clonal phylogeny of the tumor [48]. However, in this sample, the data are insufficient to distinguish between a branched phylogeny, in which the two subclones arose from independent cells within the founding clone, and a linear phylogeny, where the lower VAF subclone is descended from the higher VAF subclone. The latter case implies that all mutations in the higher VAF subclone are also present in the lower VAF subclone, as are all founding clone mutations.

### Bayesian modeling quantifies the (un)certainty of mutation clonality

**Figure 2:**
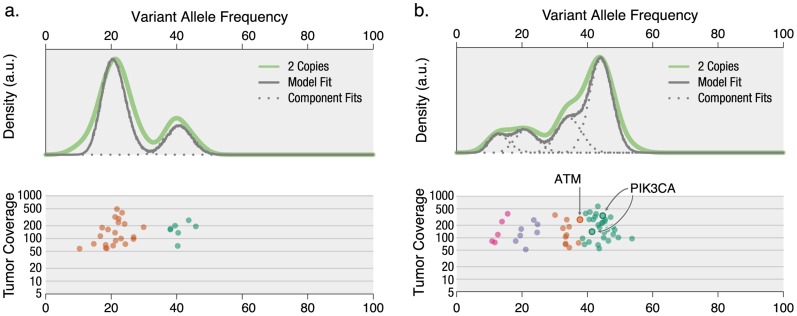
Overcoming uncertainty in sparse exome-sequencing data to determine clonal structure and mutation clonality. (a) Breast cancer sample with well-defined clones. (b) Endometrial cancer sample with overlapping clusters. *PIK3CA* mutations are strongly associated with the dominant clone (posterior probabilities 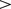
93%), whereas the clonal context of an *ATM* mutation is more ambiguous (57.8%).

Using WES to both discover variants and obtain deep read counts for defining VAFs may be an attractive, direct approach to clonality analysis [10], as it avoids the additional time and expense of WGS followed by targeted sequencing. However, while WES data captures the coding variants likely driving disease progression, their number may be insufficient to reliably infer clonal architecture, particularly for cancers with relatively low somatic mutation rates. To begin to address these considerations, we applied SciClone to whole-exome sequenced breast [49] and endometrioid carcinoma [50] cancer samples from The Cancer Genome Atlas (TCGA) project. To obtain a sufficient number of variants, we relaxed the minimum depth of coverage requirement to 50x, resulting in 29 copy-number neutral variants from the breast sample and 53 from the endometrial sample. The breast tumor has a high-VAF cluster corresponding to its founding clone as well as a subclonal cluster, with most variants occurring in the latter (Fig. 2a). The endometrial sample is more complex, with both a high-VAF cluster and three tightly-grouped and poorly-separated subclonal clusters (Fig. 2b).

Drawing inferences about mutation clonality (e.g., assessing whether mutations generally occur in the founding clone and hence are likely to be early, disease-initiating events [14] or attempting to correlate subclonal mutations with clinical outcome [10]) requires accurately and confidently assigning individual VAFs to clusters. Our variational Bayesian approach does so via “fuzzy” cluster assignments, which describe the (conditional, posterior) probability that a VAF belongs to a particular cluster (given that it belongs to one of them). In particular, the likely driver *PIK3CA* mutations in the endometrial sample are assigned confidently to the highest-frequency cluster one, with probabilities of 93.1% for the lower VAF variant and 97.1% for the higher. In contrast, the potential driver *ATM* mutation is nearly as likely to belong to cluster one (42.1% probability) as to the lower VAF cluster two (57.8% probability) to which it was “assigned” (i.e., that which maximized its posterior probability). Given the relatively few SNVs, this ambiguous assignment indicates that the data are insufficient to accurately define the clonal structure and that the separation between cluster one and cluster two may be an artifact of sparse data. This uncertainty might be resolved by increased depth of sequencing or by additional clonal markers (e.g., as discovered by WGS). Nevertheless, the strong assignments of the *PIK3CA* mutations to a cluster with average VAF near 50% suggest that, despite the relatively high level of noise in the data, they belong to the founding clone.

### Longitudinal studies refine subclonal architecture and reveal mechanisms of resistance

**Figure 3:**
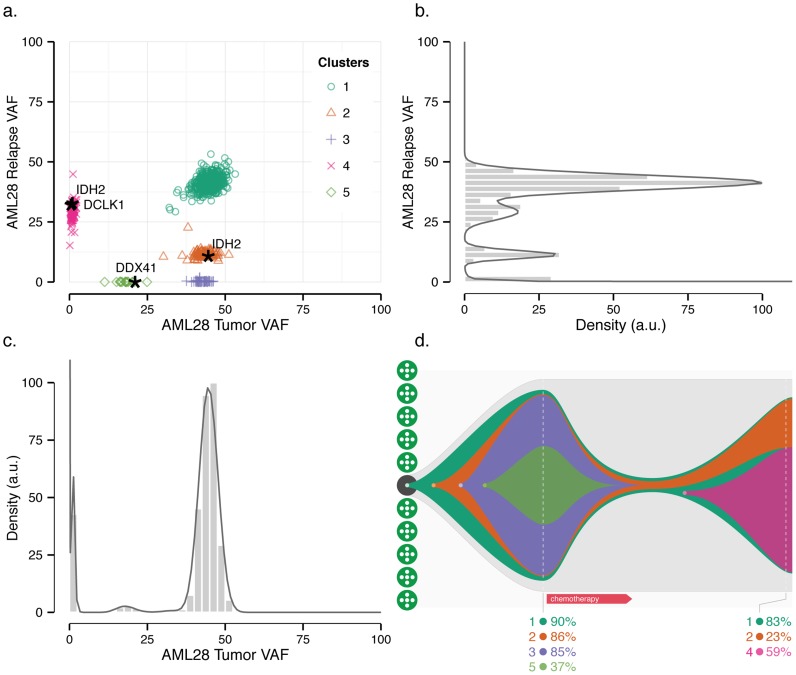
Refining subclonal architecture from longitudinal analysis of tumor/relapse pair in acute myeloid leukemia (AML). Two-dimensional analysis of tumor/relapse sample (a) dissects clusters one and four, which overlap in the relapse sample (b), and one, two, and three, which overlap in the tumor sample (c). Single-sample analyses (b and c) show histogram (rectangles) with posterior predictive densities. Several genes recurrently mutated in AML [5] are highlighted. (d) Inferred schematic of clonal evolution from a single hematopoietic stem cell, showing percentage of cells belonging to each clone (i.e., twice VAF for this nearly pure sample). Broken vertical white lines correspond to primary tumor sample (before chemotherapy) and relapse subsequent to treatment.

Tumors evolve in response to treatment, both through loss of specific mutations and acquisition of new ones. Understanding this process in the context of a tumor's clonal architecture is critically important in defining mechanisms of resistance and in informing therapeutic decisions. To better understand mechanisms of therapy resistance, we extended our method to accommodate multiple samples and applied it (Fig. 3) to samples from a primary AML tumor and post-treatment relapse occurring 26 months after chemotherapy [5]. These primary and relapse tumors were initially sequenced to depths of 26.7x and 31.5x, respectively, with subsequent capture validation providing deep read counts for all discovered variants (median depth: 753x). All variants were analyzed, as no CNAs are present in either sample.

Analysis of the primary tumor sample in isolation (Fig. 3c) suggests a simple organization consisting of a single subclone and a founding clone containing an *IDH2* R140L mutation. Mutations in this residue may play a role in oncogenesis, given their recurrence in AML [51] and resulting neomorphic enzymatic activity [52]. Hence, this clonal mutation is an attractive target for small molecule inhibitors, such as those reactive against *IDH2* R140Q [53]. However, simultaneous analysis of the relapse genome further dissects the apparently homogeneous highest-frequency cluster harboring *IDH2* R140L into three distinct subpopulations of cells (Fig. 3a): one that is effectively eliminated by chemotherapy (cluster three, average relapse VAF 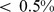), a second diminished by treatment (cluster two, average relapse VAF 11.6%), and a third largely unaffected by treatment (cluster one, average relapse VAF 41.3%). As further evidence of their high degree of overlap in the tumor sample, their respective average VAFs in the tumor are 42.7%, 43.1%, and 44.9%. The additional resolution provided by the relapse sample distinguishes these subpopulations to expose a more complex clonal architecture (Fig. 3d) and indicates that the *IDH2* R140L mutation in cluster two is subclonal. Thus, targeting it therapeutically would be unlikely to eradicate the founding clone. We do observe that the subclonal mutations in cluster five were eliminated by treatment, suggesting that it carried a lower proclivity for resistance than the surviving clones.

Remarkably, there is a second, independent *IDH2* mutation (R140W) in the relapse sample. But, as above, defining its clonality from this sample alone (Fig. 3b) is confounded by an inability to associate its VAF (32.8%) unambiguously with either the founding clone or a subclone. This uncertainty is resolved through multidimensional analysis that incorporates the tumor sample and places the mutation in cluster four. Mutations within this cluster, including *IDH2* (R140W), were either present in the primary tumor below the level of detection or are new mutations, possibly induced by cytotoxic chemotherapy [5]. In either case, they are potential drivers of disease progression.

**Figure 4:**
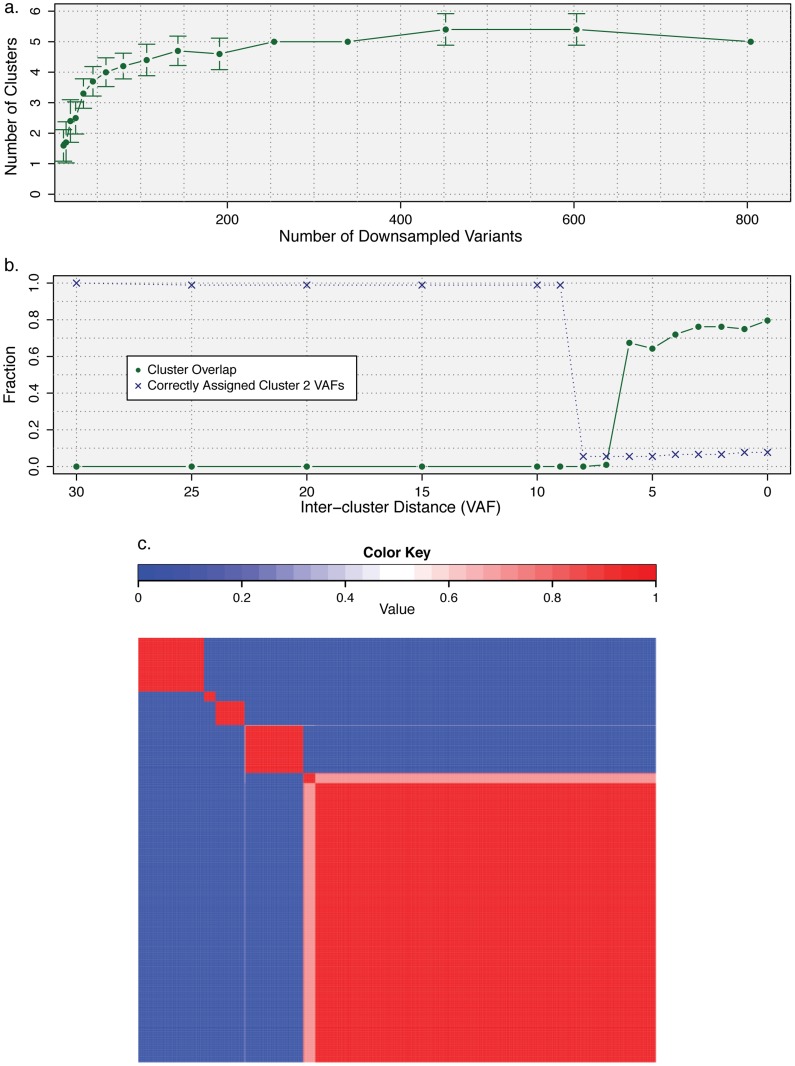
Determining stability of inferred subclones as a function (a) of number of variants, (b) of inter-cluster separation, and (c) of clustering method from AML sample. (a) A fraction of the ∼800 variants from Fig. 3 were randomly sampled and the resulting number of clusters was inferred using beta mixture modeling. Error bars represent standard deviation (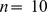). (b) Mutations from clusters one and two from the AML relapse sample were used to assess the limits of cluster separability. As the distance between the two mutation groups was varied, the resulting clusters were assessed for overlap (the fraction of the data within a single standard deviation of both clusters) and accuracy (the fraction of items that were correctly assigned to a second cluster). (c) Consensus clustering of the AML data set (Fig. 3) for number of initial clusters varied from six to 15 and clustering method varied across beta, Gaussian, and binomial mixture models for a total of 30 runs. 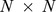 consensus matrix holds all 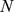 variants across both rows and columns and has been reordered so that variants belonging to the same cluster are adjacent to one another. Matrix entry 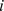, 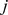 is the fraction of runs in which variant 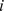 and 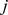 were co-clustered; entry 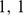 corresponds to the top-left of the matrix heat map. The narrowest neutral-colored band corresponds to a single variant alternatively classified by Gaussian mixture modeling (Fig. S2a). The larger neutral-colored band corresponds to variants alternatively classified as a sixth cluster by binomial mixture modeling (Fig. S2b).

Given the clonal complexity of this sample, we next asked how many variants were required to capture this complexity and whether we were likely to have missed additional complexity. To address these concerns, we randomly selected a subset of the original variants and performed clustering. The number of clusters inferred as a function of the number of variants analyzed is fairly constant for 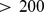 variants (Fig. 4a), whereas it drops precipitously for 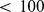 variants. As sequencing detected a total number of variants within the flat regime of this curve, we can be confident that no subclones with a higher VAF than the most infrequent cluster identified (average VAF ∼12%) were missed. Further, this suggests that ∼200 variants would have been sufficient to reveal this sample's clonal architecture. To assess the sensitivity of our approach in inferring the separation of clusters, we performed one-dimensional analysis of VAFs from relapse sample clusters one and two (Fig. 3) after varying their inter-cluster separation (Fig. 4b). While the results are sample-dependent, they indicate that clusters can be reliably distinguished if they lie greater than ∼7% away from one another.

To ensure that the inferred number and composition of clones were not overly sensitive to our computational method, we varied both the number of initial clusters and the clustering approach itself. Consensus clustering indicated that the (subjectively) correct number of clusters (five) was inferred by the variational Bayesian beta mixture modeling for a range of initial number of clusters from six to 15 (data not shown). We next used SciClone to cluster using a variational Bayesian binomial mixture model and a previously-published [40], [54] variational Bayesian Gaussian mixture model (see Text S1). Consensus clustering indicates that the results are stable for the majority of variants as both the number of initial clusters and the method (beta, binomial, or Gaussian) are varied (Fig. 4c). The few variants that clustered differently between methods (Fig. S2) were situated near cluster boundaries or between clusters. A similar effect was seen when clustering the data with PyClone (Table S1), though in this case variants along cluster boundaries tended to coalesce into independent clusters (clusters six and eight in Fig. S3): PyClone's default hyperparameter settings lead it to overdissect the founding clone. After increasing the number of iterations from 10,000 (with a burn-in of 1,000 iterations) to 100,000 iterations (with a burn-in of 10,000 iterations), PyClone results were even more similar to those obtained with SciClone, but the former still split the highest-VAF cluster into two (data not shown). Despite these differences, the results are largely consistent between SciClone and PyClone and we have increased confidence in variants that are similarly assigned by both approaches.

### Multiple biopsies reveal intratumoral heterogeneity and impact of treatment

Spatial heterogeneity complicates the analysis of solid tumors, as distinct regions of a tumor may harbor different subclonal populations [9], [13], [16]. Assaying multiple regions of heterogeneous tumors should assist in uncovering the full spectrum of mutations and subclones present in a tumor and help identify the spatial origins of subclones that give rise to therapy resistance. To investigate this effect, we analyzed two pre-treatment biopsies from the same breast tumor and added a temporal dimension by examining a single sample from the tumor collected 16 weeks after aromatase-inhibitor (AI) therapy. Mutations in the three samples had median coverage of 130.5x from deep capture sequencing.

**Figure 5:**
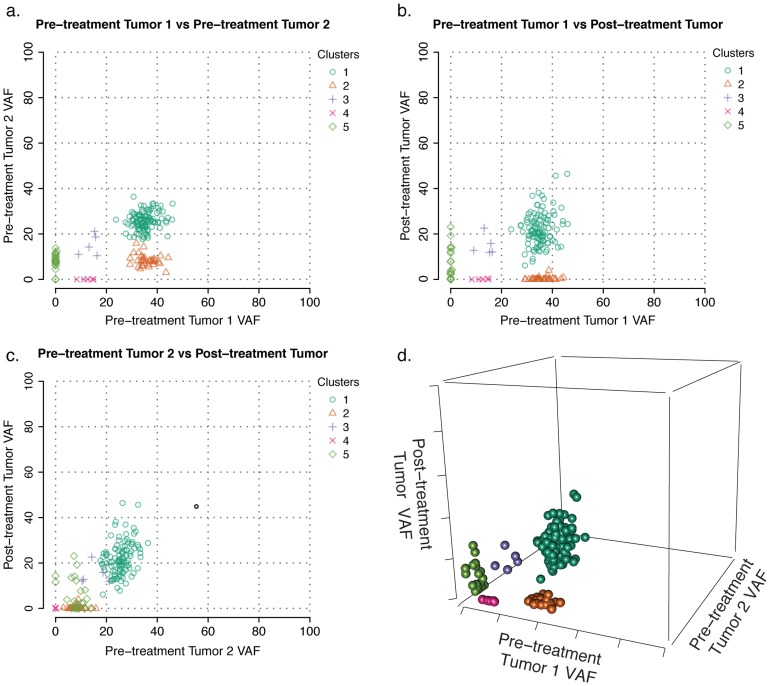
Assessing intratumor spatial heterogeneity and treatment response with multiple biopsies. Three breast tumor samples from a single individual were simultaneously analyzed: two spatially distinct samples from a primary tumor and one sample taken after aromatase-inhibitor treatment. (a–c) Two-dimensional slices and (d) still frame of the full three-dimensional interactive plot.

Three-dimensional clustering with SciClone revealed five distinct groups of mutations and a fairly low purity, resulting in reduced VAFs in all samples (Fig. 5, Movie S1). Differences between the two pre-treatment biopsies were captured in clusters four and five, containing region-specific mutations. Cluster two cannot be identified from pre-treatment tumor one alone, but the second biopsy reveals it as a distinct subpopulation of cells with higher VAF in the first biopsy (36.03% vs. 8.13%). The effect of AI therapy is revealed by inclusion of the post-treatment sample, in which clusters two and four are eliminated. These likely represent AI-responsive subpopulations of cells, though additional spatial heterogeneity leading to their apparent removal cannot be discounted. Cluster five contains mutations specific to the second biopsy; while some of the cells harboring them expanded in the post-treatment sample, others appear to have been eradicated completely. The heterogeneity in response observed in this cluster suggests that it actually encapsulates several distinct, but overlapping subclonal populations that occur at similar VAFs in the pre-treatment biopsy and are difficult to separate without additional data.

Application of PyClone to these data (Table S1, Fig. S4) reveals several significant differences. While it infers two distinct clusters from the heterogeneous cluster five, it also partitions variants in the founding clone into two clusters. This separation is likely a clustering artifact, since (1) the two clusters are merged when all of the data (in copy-altered and -neutral regions) are clustered using 34,000 iterations (data not shown) and (2) the presence of two large, *independent* clusters comprising 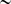
70% of the cellular population each is biologically unreasonable. The discordance between methods suggests that the limited number of variants affected require special attention.

## Discussion

Clonal heterogeneity complicates both our understanding of the biology of tumors and the design of effective treatment strategies. While an individual tumor sample provides a glimpse of this complexity, additional temporally or spatially distinct samples allow higher resolution mapping of subclonal architecture, including the isolation of drug-sensitive clones and small subpopulations driving relapse. To leverage the increasingly commonplace and cost-effective opportunities to sequence multiple samples from an individual, we developed SciClone, which scalably analyzes large numbers of samples to provide an unbiased, probabilistic dissection of a cancer's clonal landscape. To do so, SciClone employs variational Bayesian mixture modeling of beta, binomial, and Gaussian distributions. Each of these may have advantages (see Text S1) in certain situations, though our tests suggest that the beta mixture model works best in practice. We have previously used related techniques in analyzing FACS data [55] and expect them to be of general interest to those requiring methods that automatically and efficiently infer the number of clusters from high-dimensional biological data.

Application of SciClone to primary and relapse AML tumors identified subclonal populations with dramatically divergent response to conventional therapy. Such analyses are the first step towards inferring driver mutations responsible for both resistance to therapy and clonal expansion following treatment. Insight into the spatial origins of treatment response was provided by analysis of three samples from a breast tumor, two of which were obtained from distinct regions of a single tumor at the same time point.

The AML and breast cancer cases highlight an inherent limitation of bulk sequencing of tumor cells: subclonal populations cannot be distinguished if they occur at similar frequencies. Single-cell sequencing may eventually offer a solution, but will require dramatic improvements in fidelity and throughput. Using currently available data, we demonstrated that temporally or spatially distinct samples from the same tumor can be used to tease apart these overlapping subclones. This is demonstrated in AML, where the apparent founding clone in the primary tumor is dissected into two additional subclones by incorporating the relapse sample. The breast cancer samples exhibit two-fold complexity. As in the AML primary tumor, a cryptic subclone is revealed in the pre-treatment breast tumor when multiple samples are considered; in this case, from two spatially-isolated biopsies. Additionally, each pre-treatment sample exhibits a clone not detected in the other. This suggests that manipulation of the patient's tumor-derived cells (e.g., passage within culture or as mouse xenografts) may be a viable method for identifying additional subclones and predicting those with differential responses to therapy.

Our analysis of exome-sequenced cases showed that SciClone can be useful on samples with as few as 29 SNVs (Fig. 2a), but our simulations (Fig. 4a) showed that in more complex cases, such as the AML tumor/relapse pair, establishing subclonal boundaries may require two hundred or more variants and that subclones be separated by VAFs of ∼7% or more (Fig. 4b). This downsampling approach may be applied to any data set to establish a baseline sensitivity. Cases with poorly defined cluster boundaries (e.g., due to a paucity of mutations), such as the endometrial case (Fig. 2b), benefit from SciClone's probabilistic formalism. In particular, by assigning an *ATM* mutation similar probability of belonging to the founding clone and a subclone, SciClone reflected the lack of certainty inherent in the data and indicated that their sparsity may poorly characterize the tumor's clonal diversity. The sensitivity of any clustering method in dissecting clonal boundaries is dependent on cluster overlap, which we have characterized via the “uncertainty” of their probabilistic assignments (Refs. 45 and 46 and Materials and Methods). An additional, qualitative means of detecting high-confidence variant/cluster assignments involves taking the consensus, or intersection, across clustering methods (Fig. 4c). Confidence in detecting all major subclones increases with the number of variants, including passenger mutations more likely to be missed by exome sequencing. Thus, WGS followed by deep validation sequencing is most likely to capture the full spectrum of mutations and yield high-quality characterization of subclonal entities.

Next-generation sequencing of variants within copy-number neutral regions of autosomal chromosomes leads to a straightforward interpretation of the inferred VAFs as half the cellular frequency harboring the corresponding variant. Because of the widespread availability of variants to serve as clonal markers and the relative reliability of their bioinformatic analysis and quantification, our initial clonality analyses have focused on SNVs. Nevertheless, other genomic events have been used to identify clonal dynamics. For example, the alternate “waxing” and “waning” of subclonal CNAs has been observed in multiple myeloma [17], [33]. However, the analysis and discovery of CNAs pose several challenges for clonality: (1) Cancer types may be described hierarchically in terms of their propensity to elicit either mutations or copy number changes [56]. For mutation-dominated cancer types, such as the cytogenetically-normal AML analyzed here, few CNA events may be available for analysis. The converse does not apply: since SNVs accumulate with age [4], an abundance of SNV clonal markers are expected in all malignant, as well as in non-malignant, tissues. Given the density of SNVs, clonality analyses that rely solely on them may well capture the full clonal architecture, while missing specific (copy number) events of pathogenic interest; clonality analyses relying solely on copy number events are likely to miss both. (2) There is no digital readout of CNAs, rather observed copy number reflects the admixture of subclonal populations and is a (linear) combination of the copy number state of each subclone, weighted by the fractional subpopulation of the subclone. In principle, the correctness of the analysis requires the simultaneous inference of this admixture and the number of copies (0, 1, 2, 3, or greater) of each chromosomal segment in each subclone. Though such an analysis would infer the clonal hierarchy directly, rather than the clusters of variants that serve to identify them as in a SNV-based analysis, inference in the latter case is simplified since there are fewer mutational states (presence or absence, at least of the vast majority of variants, which are heterozygous) and the correctness of inferring one cluster is independent of a second cluster.

For these reasons, we prefer to overlay CNA events on the higher confidence copy-number neutral SNV VAFs. Incorporating such events is important: (1) to include SNVs from CNA regions, which are especially likely to be involved in disease, and (2) to ameliorate the loss of SNVs from copy-number neutral regions occurring as the number of genomic regions perturbed by CNA (in some sample) increases with the number of samples analyzed. Accommodating these events may be accomplished: (1) by determining the fractional population harboring the event (as in Fig. S1) or (2) by adjusting a SNV's VAF based on it's inferred copy number states across subclonal populations. One approach to the latter involves inferring copy number states from the B-allele frequencies of germline SNPs (e.g., using ASCAT [34] or APOLLOH [39]) and phasing these to somatic variants (e.g., by detecting a single sequencing read spanning both) to impute subclone-specific copy numbers to each variant [6]. After adjusting the SNV VAFs, they could be clustered by SciClone in a manner completely analogous to the analysis of unadjusted VAFs (using the beta or Gaussian mixture model approaches). We are currently pursuing this approach.

MCMC techniques, such as PyClone [11], [30], offer an alternative approach to clustering variants. However, our comparisons of SciClone and PyClone (Table S1) reinforce the computational inefficiencies of MCMC approaches relative to variational Bayesian techniques [40] and show that SciClone is between one and two orders of magnitude faster. SciClone inherits the simple variational Bayesian (computational) convergence condition of monitoring monotonic changes in a lower bound (see Text S1). While this approach may converge to a local extremum, more subtlety is required to ensure the (theoretical) asymptotic convergence to the global extremum guaranteed by MCMC, e.g., monitoring variance within a Markov chain relative to variance between independent Markov chains [41]. PyClone provides no direct facilities to monitor convergence. Regardless, the theoretical convergence properties of MCMC seem unjustified given the involved computational overhead for a clustering application, such as clonality detection, where error estimates of the parameters are of marginal interest.

SciClone has already contributed to the understanding of biological mechanisms underlying cancer and has the potential for increased utility with the advent of clinical sequencing. Towards this end, we are developing methods that cross-reference the clonal status of specific mutations with databases of targeted therapeutics. As an example, the Drug-Gene Interaction Database [57] identifies three genes in the AML sample as as potentially druggable: (*DRD2*, *KCNQ2*, and *P2RY2*). The fact that each of these mutations lies in a subclone complicates their interpretation, and suggests that careful study is needed to understand how specific subclonal populations respond to different therapeutics. While clinical decisions of which (sub)clones to target and how remain controversial, it is clear that making these decisions will require accurate assessment of clonal architecture using tools such as SciClone.

## Materials and Methods

### Variational Bayesian mixture modeling of beta distributions

A VAF 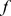 is defined with respect to the number of reads, 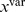, supporting the variant allele and the number of reads, 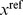, supporting the reference (or non-major-variant, in the case of multiple variants) allele: 
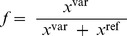
. Our previous method [14] considered variants in a *single* sample and modeled the probability of a VAF 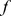 belonging to cluster 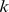 as
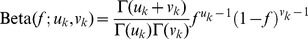
where 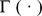 is the gamma function. Here, we extend this to the case with 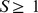 samples by defining the 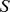-vector 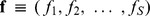, whose 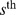 component, 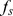, is the VAF of that variant in the 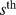 sample. We make the assumption that, *within a cluster*, the VAFs are independent across samples, so that the cluster may simply be modeled as
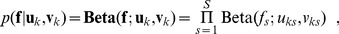
(1)where 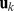 and 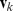 are the 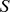-vectors whose 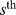 components are 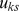 and 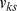, respectively. This implies only that *within* a cluster there is no correlation between samples. The validity of this assumption is indicated by the visually-observed orthogonality of the VAF principal component axes to the coordinate (i.e., sample) axes. We have rarely, if ever, seen evidence for such intra-cluster correlation. Nevertheless, this assumption may be relaxed through use of a mixture of multivariate Gaussian distributions (see Text S1), each of which has a full-rank covariance matrix.

In considering a mixture of 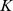 (multi-dimensional) beta components [Eq. (1)], we introduce a 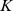-dimensional latent (or unobserved) binary random variable 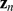 indicating whether VAF 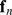 does (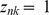) or does not (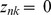) belong to component 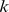 and satisfying a 1-of-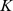 representation in which 
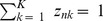
. The marginal probability 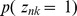 that a VAF belongs to component 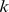 is given by its mixing coefficient 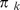,
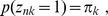



subject to the probabilistic constraints
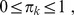


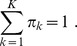



Given the 1-of-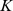 representation of 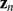, this may be written as
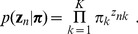
(2)


Similarly, the conditional distribution 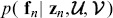 that a VAF 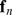 arises from the mixture may be written
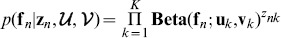
(3)in terms of the shape parameter vectors 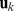 and 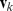 of the 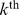 beta component, with aggregate parameters 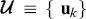 and 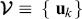.

To accommodate binomial and Gaussian mixture models in addition to the beta mixture model, we introduce abstract notation used below to define quantities (e.g., 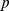-values) independently of the concrete representation of likelihoods and posterior distributions. We begin by defining abstract parameters 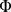, which differ according to the model, i.e., beta, binomial, or Gaussian. For example, 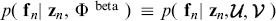, with 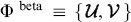. Further, while the Gaussian mixture model is also a function of VAFs 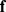, the binomial mixture model is defined with respect to the variant and reference count vectors, 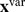 and 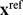, respectively. To abstract away these details, we use the notation 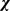 to denote the VAFs 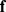 of a beta or Gaussian mixture model or the counts 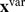 and 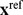 of a binomial mixture model, when convenient. In particular, 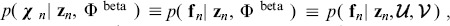, while 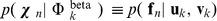, with 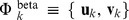.

Eqns. (2) and (3) extended across the entire set 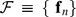 of VAFs (or, more abstractly, 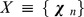 of data) and their associated latent variables 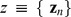 are combined to express the complete-data (i.e., including the latent variables) likelihood
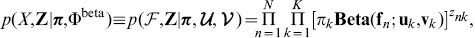
(4)which may be summed over 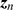 to give the incomplete likelihood
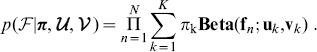



These equations could be used to fit the beta parameters using expectation maximization (EM) or Markov chain Monte Carlo (MCMC) techniques.

We instead use a previously described [43], [44] variational Bayesian approach [40], [42], [54], [58], [59] to modeling a mixture of beta distributions. The general variational Bayesian theory and its application to mixture modeling are described in depth in several excellent references [40], [42], [54], [58], [59]. To establish consistent notation, we provide an abridged, but self-contained, introduction to this general theory and to its application to Gaussian [40], [54], [58], [59] and binomial mixture models in Text S1. Here, we summarize its application to beta mixture models to provide sufficient context for its use in and extension for clonality analysis. For full details of this derivation, the reader is referred to the original references [43], [44].

Variational Bayesian beta mixture modeling approximates the posterior distribution, 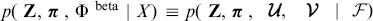, over the model parameters 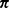, 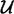, and 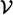 and the latent variables 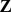 with a distribution 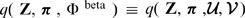. The form of this approximate distribution is a consequence of choice of prior distribution, whose product with the likelihood [Eq. (4)] defines the posterior 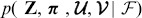 according to Bayes' theorem, and of the mild and standard [40] assumption that the latent variables 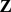 factorize from the model parameters, i.e., 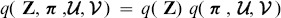. This further simplifies, without assumption, to 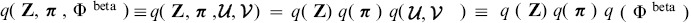. Finally, the authors assume the 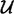 and 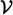 variables are independent and factorize to ultimately give 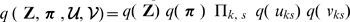.

Ma and Leijon used four synthetic one-dimensional data sets (Fig. 4 of Ref. 43), including two with highly overlapping beta distributions, to demonstrate the high accuracy of this method despite its assumption that the parameters of the beta distribution are independent. Fan et al. [44] additionally analyzed six three-dimensional data sets and similarly found that accuracy was not adversely effected by this factorization approximation (Table I of Ref. 44). Our own extensive simulation results further support these findings. We generated data sets by sampling a mixture of beta distributions in one, two, or three dimensions and having between two and five clusters (100 data sets for each dimensionality/number of clusters pair). Fig. S5 shows the concordance (i.e., fraction of correctly assigned items) between the clustered and known results for each simulated data set. The average concordance is 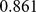, 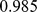, and 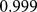 in one, two, and three dimensions, respectively. Performance increases with dimensionality as the clusters become increasingly separated. This sparsity may be quantified by the minimum cluster self-overlap (see below). Data sets having a relatively small minimum cluster self-overlap have a relatively large overlap *between* clusters, which leads to uncertainty and degrading performance.

The prior distributions are generally selected to be conjugate to the likelihood for analytic convenience (e.g., see the derivations of the variational Bayesian Gaussian and binomial mixture models in Text S1). While a conjugate prior to the beta likelihood is formally available, its use would lead to an analytically intractable integration [43]. Therefore, Ma and Leijon [43] instead propose use of the following prior distribution
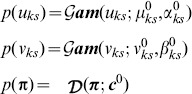
(5)where 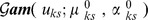 and 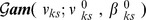 are gamma distributions
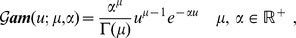
and 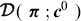 is the Dirichlet distribution
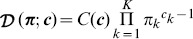
over proportions 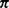, with the normalizing constant 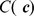


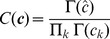
and
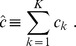



The parameters of the approximate posterior distribution are now determined by iteratively minimizing the Kullback-Leibler divergence, a measure of the difference, between it and the posterior distribution, following the general prescription of variational Bayesian inference (see Text S1). The authors make a non-linear approximation to an expectation value arising during the iterative procedure so that the resulting, approximate posterior distribution has the form of a gamma distribution, despite the fact that the above gamma prior distribution is not conjugate to the beta likelihood. Significantly, the authors show that this additional approximation can be used to minimize the original, desired Kullback-Leibler divergence between the posterior distribution and the approximate, non-gamma posterior distribution. This results in the approximate posterior distribution
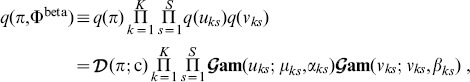
(6)where 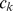, 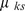, 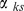, 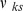, and 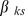 are defined in Eqns. 47–51, respectively, of Ref. 43. These parameters are updated from the corresponding initial hyperparameter values 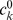, 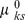, 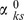, 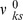, and 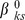 as in a traditional EM iterative algorithm. It will also be convenient to define the posterior density with respect to the 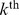 component
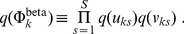
(7)


### Probabilistic and hard cluster assignments

Variational Bayesian mixture models provide probabilistic assignments of variant 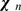 (i.e., a VAF 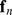 for beta or Gaussian mixture models or variant counts 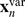 for a binomial mixture model) to cluster 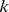 according to the posterior probabilities 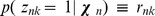. The 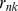 act as “responsibilities” and satisfy 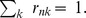 In the case of the beta mixture model, the 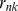 are defined by Eqns. 31 and 32 of Ref. 43. A more general derivation is provided in Text S1, along with specific calculations for binomial and Gaussian mixture models.

For visualization purposes, for example, we occasionally transform these probabilistic assignments into hard assignments, which assign 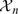 to one and only one cluster 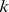 according to
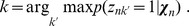



### Posterior predictive density

The posterior predictive density gives the probability of a new (i.e., unobserved) variant, 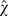, given the observed data 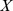


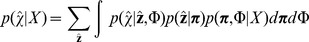
and all possible assignments 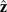 of that variant to a cluster. Evaluating the sum over 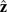, making use of Eq. (2), gives
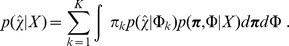



We next approximate the true posterior distribution, 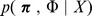, with the variational approximation 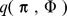 to give
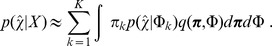



Since for all mixture models considered in this manuscript 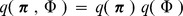, with 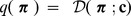 and
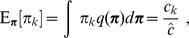
this evaluates to
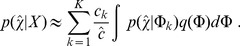
(8)


Ma and Leijon [43] assumed that 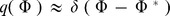, where 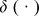 is the Dirac delta function and 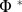 are the converged parameter values, i.e., that the posterior distribution has negligible probability when any of the parameters differ from their converged values. In this case,
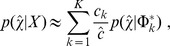
which may be efficiently evaluated. We instead use Eq. (8), which avoids any assumption on the approximate posterior distribution. In the case of binomial and Gaussian mixture models, Eq. (8) may be evaluated analytically. In the case of a beta mixture model, for which 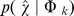 is given by Eq. (1) and 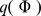 is given by Eq. (7), we instead resort to numerical integration, evaluating data sampled from Eq. (7) via Eq. (1).

### Prior initialization

We choose hyperparameters resulting in prior distributions sufficiently broad to ensure that the number of clusters and their posterior parameterization are determined primarily from the data rather than from prior assumptions. In particular, following Ma and Leijon [43], we choose 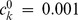 for all 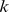. We also choose 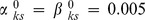 and 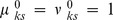 for all 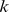. Given the latter choice, the gamma distributions 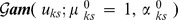 and 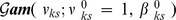 collapse to exponential distributions. The resulting variances of these distributions, e.g., 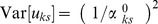, are large given our choice of hyperparameters and hence provide a broad prior.

We initialize the 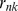 according to the hard assignments computed by 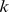-means (provided in the R stats package and using default parameters, except with 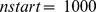 and 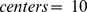). We initialize the parameters 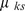, 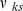, and 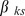 to their respective hyperparameter values 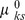, 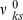, and 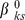. Finally, we initialize the 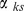 such that the expected means of the cluster centers, 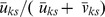, with 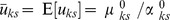 and 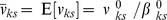, are set to the values returned by 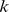-means. We then perform the variational E step (i.e., calculate the expectations immediately following Eq. 51 of Ref. 43) *without* updating the 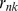, followed by the variational M step to update the parameters 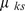, 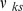, 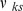, 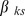, and 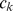 (via Eqns. 47–51 of Ref. 43). For the AML28 data set, this initialization results in the clusters shown in Fig. S6a. Initialization is followed by iteratively applying the variational E step (including updating the 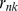) and M step. To avoid undefined behavior in evaluating the beta distribution, we shift VAFs at zero or one by 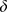 or 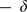, respectively, with 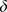 equal to machine precision.

### Cluster pruning and outlier detection

Variational Bayesian mixture modeling generally discards clusters that do not contribute to the model, as determined by the data and strength of the prior distribution. Specifically, following convergence of the variational iteration and hard assignments of variants to clusters, we remove any clusters having less than the larger of three variants or 0.5% of 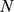, the total number of variants, assigned to them, a condition similar to our earlier approach [14]. If clusters are removed, the algorithm is again executed until convergence. For the beta mixture model, convergence is achieved when the absolute difference between all 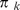 across consecutive iterations is less than 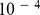. This condition differs slightly for binomial and Gaussian mixture models (see Text S1). The minimum cluster membership is motivated by the requirement of needing at least two proportions to fix the two degrees of freedom, 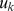 and 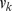, of a beta distribution. More intuitively, clustering is effectively a separation of intra- and inter-cluster distances. Defining an intra-cluster distance requires at least two items be assigned to that cluster.

To be conservative in our assessment of subclonality, we require clusters be well separated. Previously [14], we used a condition on overlapping cluster standard error of the means to detect and remove overlapping clusters. Here, we instead adopt a quantitative notion of cluster overlap [45], [46], in which overlap between clusters 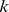 and 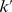 results in uncertain assignments of some variants, causing them to have appreciable 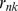 *and* 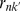. This in reflected in a large relative (to the “size”, 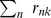, of cluster 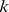) cluster overlap
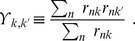



Minimizing this quantity for all 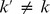 is equivalent to maximizing the “self-overlap” of cluster 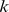 with itself,
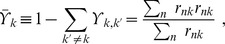
which satisfies, 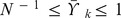. Hence, we remove any cluster having a 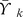 less than a threshold 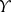. Overlap between (independent) clusters will be more likely in lower dimensional problems; hence, to determine a dimensionality-dependent 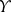 we clustered simulated data sets by sampling a mixture of beta distributions in one, two, or three dimensions and having between two and five clusters. Average concordance (across data sets of a given dimensionality) between the clustered and known results (in terms of fraction of correctly assigned items) was stable for a wide range of 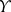 within each dimension: 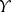 in the range of 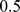 to 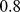 achieved the maximal concordance (of 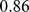) in one dimension, 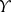 in the range of 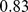 to 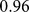 achieved a concordance of 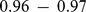 in two dimensions, and 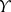 in the range of 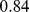 to 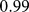 achieved a concordance greater than or equal to 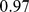 in three dimensions. Intuitively, we anticipate that the probability of clusters overlapping scales inversely with the number of dimensions. Hence, we define 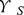 for an 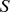-dimensional problem as 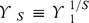, where 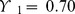 was selected so that 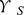 passes through the above optimal regions defined by the simulation. Namely, 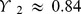 and 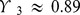. Results for these settings of 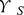 across all simulated data sets are shown in Fig. S5.

We detect outliers using a more formal approach than our previous method [14], by calculating the 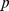-value of a variant with the respect to the cluster to which it has been assigned (via a hard assignment). If the probability of the variant belonging to that cluster is less than 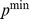, the variant is removed from the analysis. The default used in this manuscript is 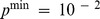 (which is *not* corrected for multiple testing). The 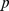-value of a variant 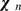 is calculated with respect to the predictive posterior distribution [Eq. (8)] as
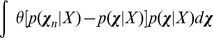
where 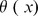 is the Heaviside step function with 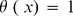 for 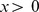 and 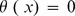 for 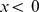. In the case of beta mixture models, this integral is evaluated numerically by sampling from the predictive posterior distribution and then evaluating sampled variants with that distribution, which again involves numerical integration. For computational efficiency, we only calculate this integral for variants likely to be outliers, which we heuristically define as variants whose VAF 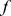 in each sample 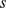 lies outside of the narrowest interval containing 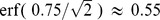 of the fluctuation in the mean of cluster 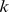. This interval 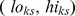 is determined as the narrowest such interval satisfying
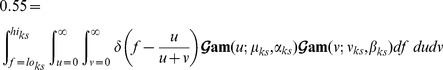
and involves integrating the mean, 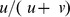, with respect to the posterior distribution.

Several iterations of the AML28 data set following the 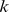-means initialization (above and Fig. S6a) are shown in Figs S6b and c, with the complete run shown in Movie S2.

### Variant detection and copy number calling

Sequencing, alignment, and variant calling were performed as previously described [5]. Somatic copy number events were detected using copyCat (http://github.com/chrisamiller/copycat/). Copy-number neutral LOH was detected using VarScan 2 [38] and filtered to retain regions with 95% LOH and at least 10 sites.

### PyClone

PyClone version 0.12.3 was downloaded from http://compbio.bccrc.ca/software/pyclone/. VarScan 2-detected regions of LOH were excluded from analysis. Copy number events detected by copyCat were quantized and passed to PyClone as major_cn, with minor_cn set to zero; additionally, PyClone was run with ––var_prior total_copy_number, since allele-specific copy number calls were not provided. PyClone clustering used the beta-binomial mixture model. Initially, we attempted to cluster using 10,000 iterations and 1,000 burn-in iterations, as suggested by the authors (Ref. 30 and https://bitbucket.org/aroth85/pyclone/wiki/Tutorial). However, these parameters yielded discordant clusterings across three runs. Therefore, we varied the number of total iterations (and additionally varied the number of burn-in iterations to be 10% of the total iterations) and for each configuration assessed concordance across three independent runs. The authors have suggested similar approaches based on visual inspection of convergence across randomly-initialized runs [30]. We choose the number of iterations at which the concordance across the three runs stabilized. These are given in Table S1. Concordance was evaluated for each of the three pairs and was calculated as the maximal fraction of items assigned to the same cluster across permutations of the cluster labels of one of the two runs being compared.

### THetA

THetA version 0.51 was downloaded from http://compbio.cs.brown.edu/projects/theta/. After failing to successfully run the program on the complete set of copy number events in the MM sample, we selected seven copy number regions, representing neutral, amplified, and subclonally deleted chromosomes, and ran THetA as described in the manual (parameters: –n 3 –k 4 –m 0.10 ––NUM_PROCESSES 2). The resulting population frequencies and copy number assignments were used to infer the VAF at which a SNV in that region would appear. These sites were added to the list of SNV inputs to SciClone and clustered with default parameters.

## Supporting Information

Figure S1 **Integration of copy number-derived subclonal information from THetA.** THetA was used to detect clonal and subclonal copy-number events in a multiple myeloma sample, then converted to pseudo-VAFs and co-clustered with SNV data using SciClone. CN-derived points are highlighted in yellow. The leftmost two CN events are single points and the rightmost point consists of six overlapping points.
(TIF)

Figure S2 **Detecting ambiguous or low-confidence associations between a variant and clone from inconsistent assignments across clustering methods.** Clonal dissection of AML sample (Fig. 3) based on (a) Gaussian or (b) binomial variational mixture modeling. Beta mixture modeling (Fig. 3) differs from Gaussian mixture modeling in the single variant highlighted by arrow in (a) and from binomial mixture modeling in the separation of cluster two from cluster one in (b).
(TIF)

Figure S3 **Confirming subclonal AML populations using an independent method.** PyClone largely recapitulates subclonal architecture inferred by SciClone (Fig. 3), though the parameter settings used here (default hyperparameters to beta-binomial mixture, with 10,000 iterations, and a burn-in of 1,000 iterations) overdissect the founding clone.
(TIF)

Figure S4 **Confirming subclonal breast tumor populations using an independent method.** PyClone clustering of variants in copy-number neutral regions is similar to that obtained by SciClone (Fig. 5), though the former partitions the variants spread along the pre-treatment tumor 2 axis (clusters 1 and 2), as well as those belonging to the founding clone (clusters 7 and 9). Subpanels (a–c) correspond to two-dimensional slices in Fig. 5 of three breast tumor samples (two spatially distinct samples from a primary tumor and one sample taken after aromatase-inhibitor treatment).
(TIF)

Figure S5
**Assessing concordance between known and clustered results.** Beta mixtures having two to six components were sampled in (a) one, (b) two, or (c) dimensions and clustered. Concordance is the fraction of data points correctly clustered; the highest concordance resulting from a permutation of the cluster labels is reported. Reported self-overlap is the minimum reported over any cluster, i.e., 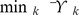. Self-overlap is shifted by 0.1 in the plots for visual purposes to avoid obscuring concordance.(TIF)

Figure S6
**Converging to clustering solution using variational Bayesian beta mixture model.** 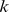-means initialization (A) of AML sample (Fig. 3) and results following second (B) and fourth steps (of six) in iteration (C).(TIF)

Movie S1 **Interactive, three-dimensional clustering of three breast tumor samples from a single individual (see** **Fig. 5d** **).**
(MP4)

Movie S2 **Movie of convergence of AML sample clustering (see** **Fig. 3** **and Fig. S4).**
(SWF)

Table S1 **Execution time of SciClone (Variational Bayes) and PyClone (MCMC).**
(PDF)

Text S1 **Supplemental methods and discussion.**
(PDF)
