# Supplementary figures and images for: SciClone: Inferring Clonal Architecture and Tracking the Spatial and Temporal Patterns of Tumor Evolution
Source: PLoS Comput Biol. 2014 Aug 7;10(8):e1003665. doi: 10.1371/journal.pcbi.1003665 (PMC4125065; doi:10.1371/journal.pcbi.1003665)

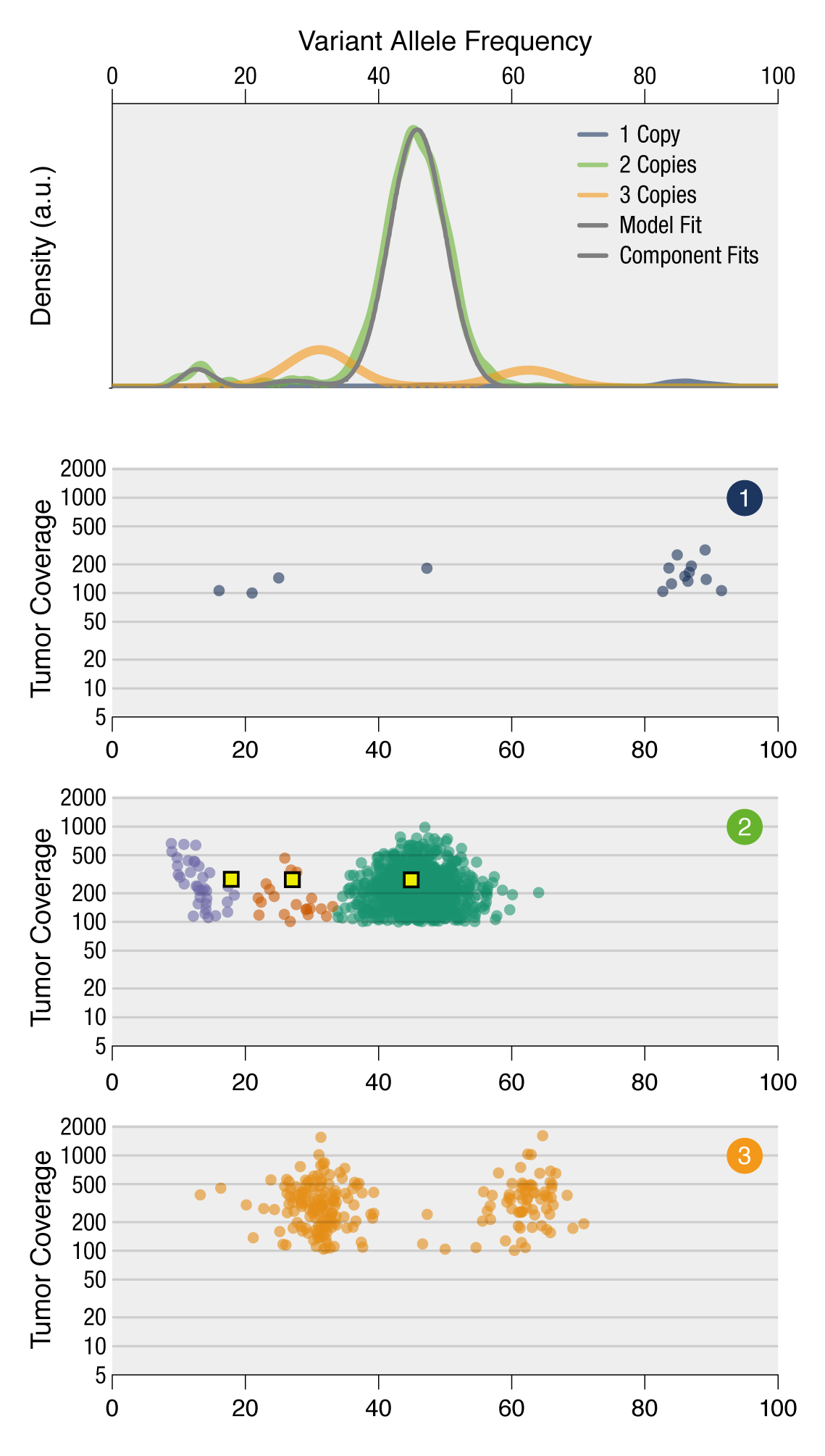

Supplement: Figure S1 — Integration of copy number-derived subclonal information from THetA. THetA was used to detect clonal and subclonal copy-number events in a multiple myeloma sample, then converted to pseudo-VAFs and co-clustered with SNV data using SciClone. CN-derived points are highlighted in yellow. The leftmost two CN events are single points and the rightmost point consists of six overlapping points. (TIF) [file pcbi.1003665.s001.tif]

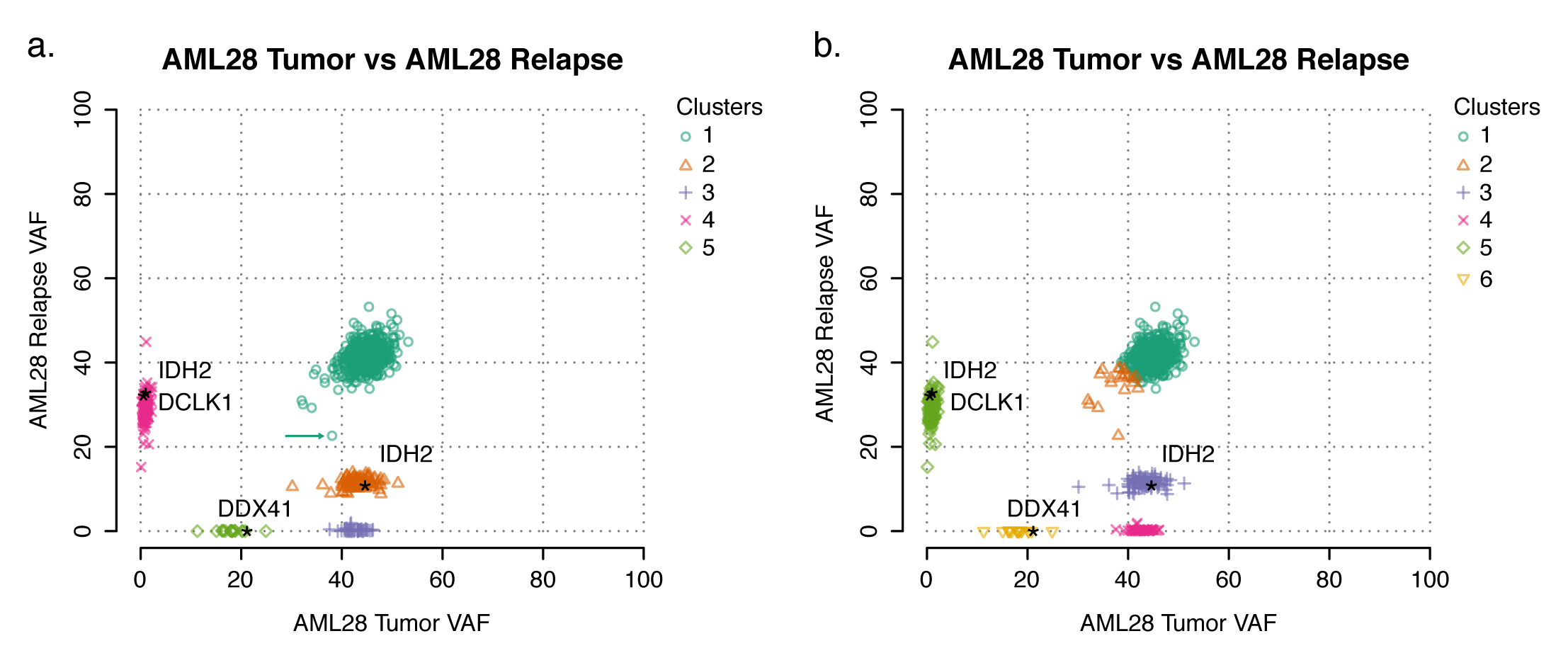

Supplement: Figure S2 — Detecting ambiguous or low-confidence associations between a variant and clone from inconsistent assignments across clustering methods. Clonal dissection of AML sample (Fig. 3) based on (a) Gaussian or (b) binomial variational mixture modeling. Beta mixture modeling (Fig. 3) differs from Gaussian mixture modeling in the single variant highlighted by arrow in (a) and from binomial mixture modeling in the separation of cluster two from cluster one in (b). (TIF) [file pcbi.1003665.s002.tif]

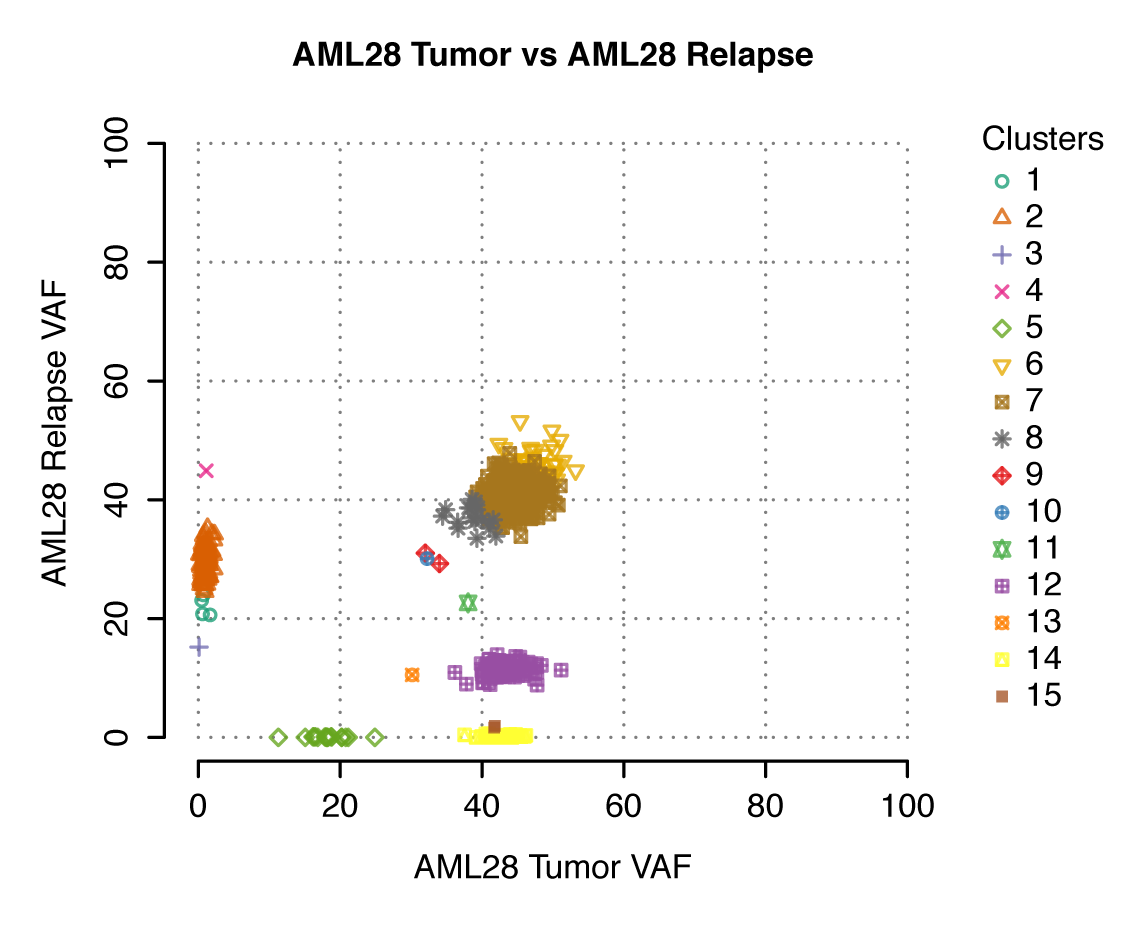

Supplement: Figure S3 — Confirming subclonal AML populations using an independent method. PyClone largely recapitulates subclonal architecture inferred by SciClone (Fig. 3), though the parameter settings used here (default hyperparameters to beta-binomial mixture, with 10,000 iterations, and a burn-in of 1,000 iterations) overdissect the founding clone. (TIF) [file pcbi.1003665.s003.tif]

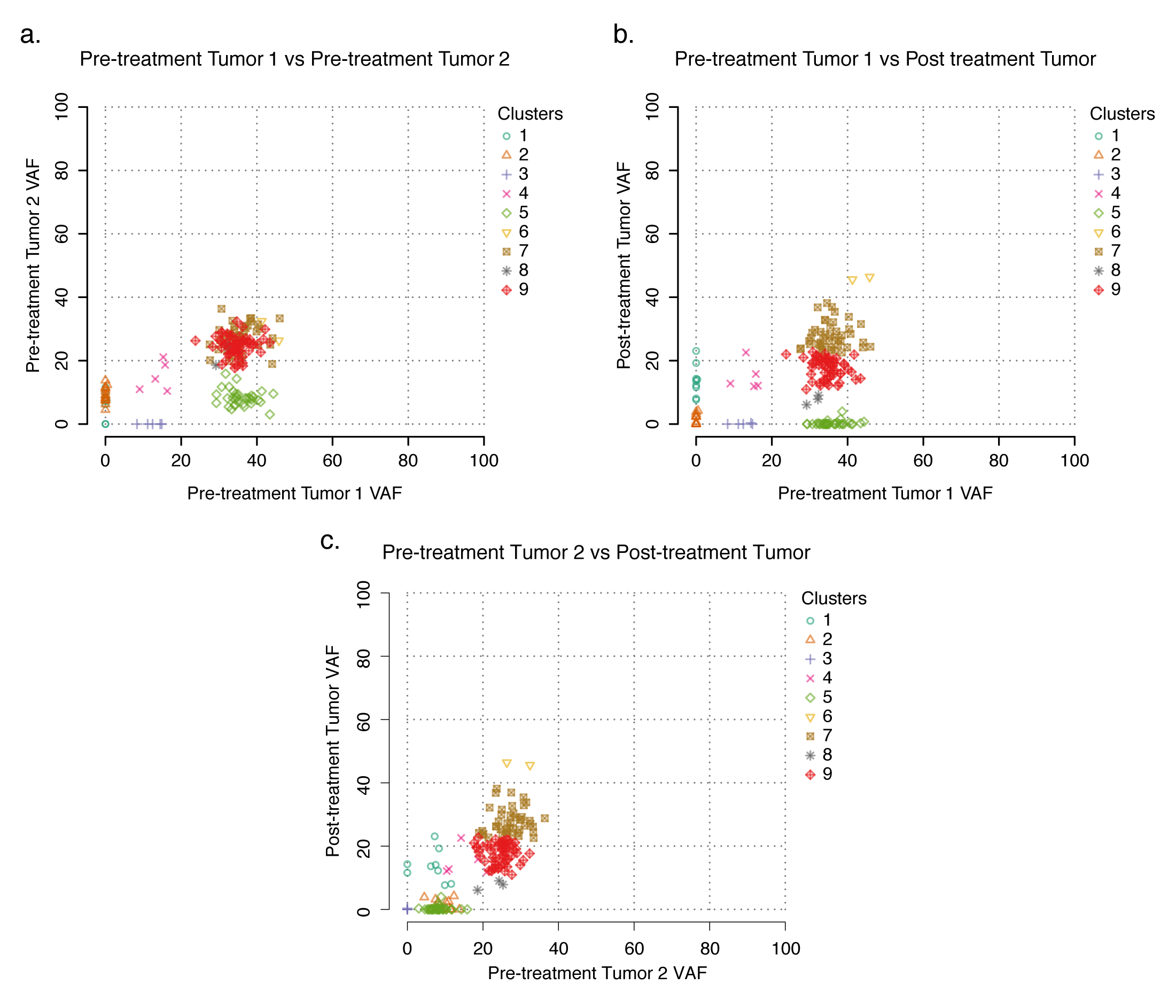

Supplement: Figure S4 — Confirming subclonal breast tumor populations using an independent method. PyClone clustering of variants in copy-number neutral regions is similar to that obtained by SciClone (Fig. 5), though the former partitions the variants spread along the pre-treatment tumor 2 axis (clusters 1 and 2), as well as those belonging to the founding clone (clusters 7 and 9). Subpanels (a–c) correspond to two-dimensional slices in Fig. 5 of three breast tumor samples (two spatially distinct samples from a primary tumor and one sample taken after aromatase-inhibitor treatment). (TIF) [file pcbi.1003665.s004.tif]

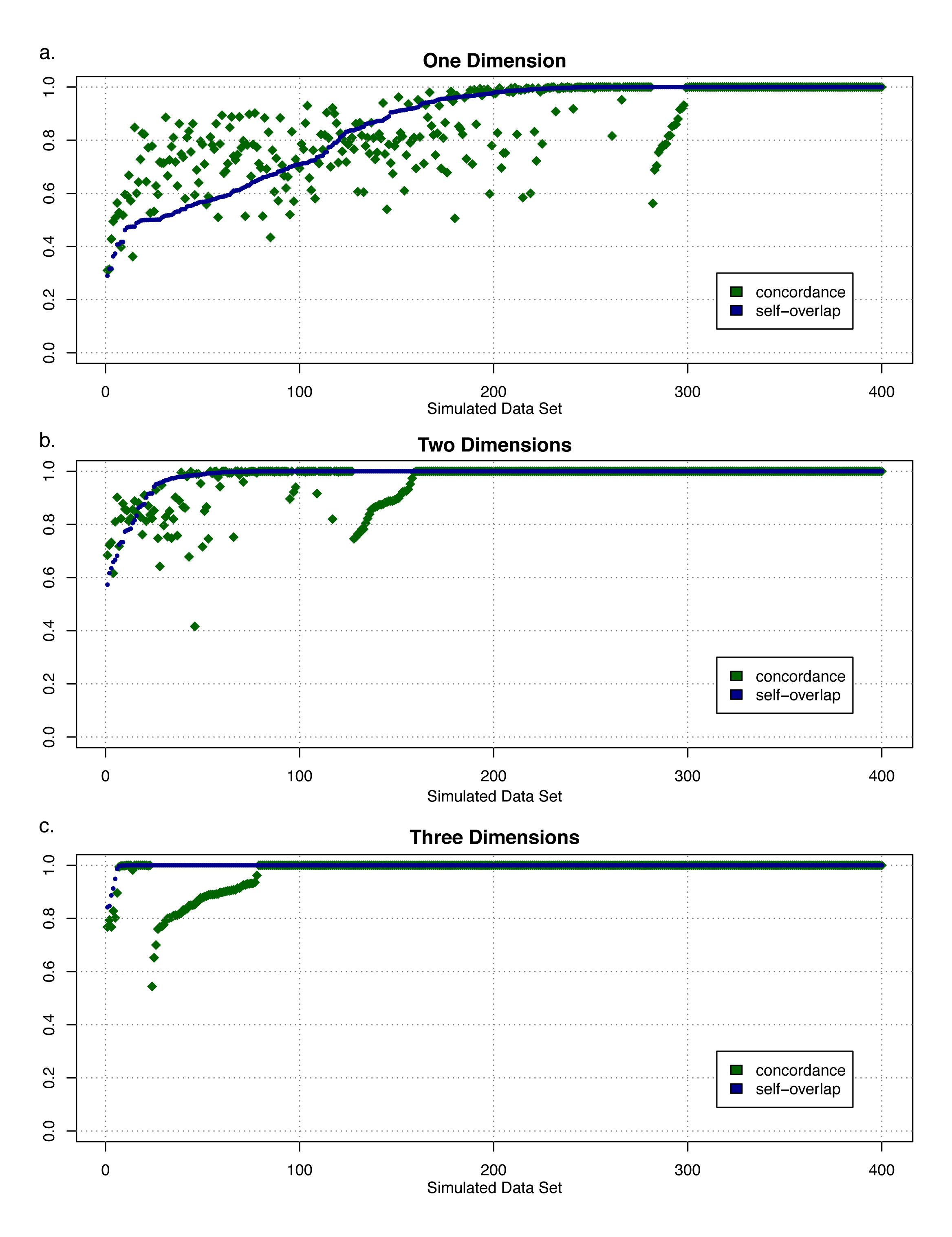

Supplement: Figure S5 — Assessing concordance between known and clustered results. Beta mixtures having two to six components were sampled in (a) one, (b) two, or (c) dimensions and clustered. Concordance is the fraction of data points correctly clustered; the highest concordance resulting from a permutation of the cluster labels is reported. Reported self-overlap is the minimum reported over any cluster, i.e., . Self-overlap is shifted by 0.1 in the plots for visual purposes to avoid obscuring concordance. (TIF) [file pcbi.1003665.s005.tif]

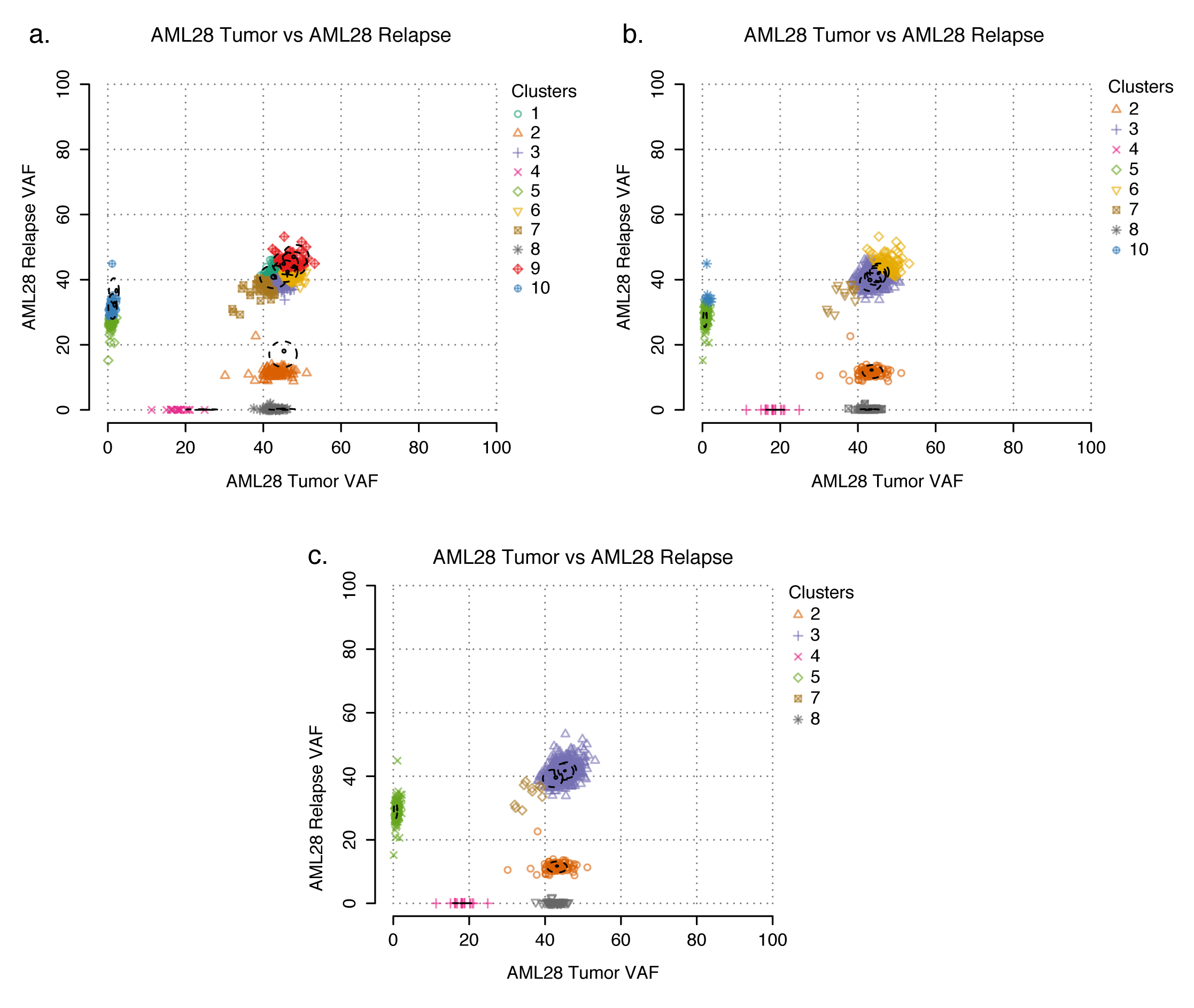

Supplement: Figure S6 — Converging to clustering solution using variational Bayesian beta mixture model. -means initialization (A) of AML sample (Fig. 3) and results following second (B) and fourth steps (of six) in iteration (C). (TIF) [file pcbi.1003665.s006.tif]
