# Supplementary material for: SciClone: Inferring Clonal Architecture and Tracking the Spatial and Temporal Patterns of Tumor Evolution
Source: PLoS Comput Biol. 2014 Aug 7;10(8):e1003665. doi: 10.1371/journal.pcbi.1003665 (PMC4125065; doi:10.1371/journal.pcbi.1003665)
Supplement: Table S1 — Execution time of SciClone (Variational Bayes) and PyClone (MCMC). (PDF) [file pcbi.1003665.s009.pdf]

TABLE S1: Execution Time of SciClone (Variational Bayes) and PyClone (MCMC)

Data Set	SciClone	PyClone		
	Elapsed Time (sec)[a]	Iterations	Burn-in	Elapsed Time (sec)[a b]
MM[c] (Fig. 1)	137.439 (20.053)	20,000	2,000	4,048.451 (207.430)
AML[d] (Fig. 3)	49.837 (2.867)	20,000	2,000	14,267.76 (303.021)
BRC[c] (Fig. 5)	158.856 (9.946)	22,000	2,200	2,265.293 (68.893)

[a]Mean elapsed time across ten runs. Standard deviation in parentheses.
[b]Elapsed times include analysis (`PyClone analyze`) and clustering (`PyClone cluster`).
[c]Analysis restricted to variants in copy-number neutral regions.
[d]All variants are in copy-number neutral regions.
