# Supplementary material for: SciClone: Inferring Clonal Architecture and Tracking the Spatial and Temporal Patterns of Tumor Evolution
Source: PLoS Comput Biol. 2014 Aug 7;10(8):e1003665. doi: 10.1371/journal.pcbi.1003665 (PMC4125065; doi:10.1371/journal.pcbi.1003665)
Supplement: Text S1 — Supplemental methods and discussion. (PDF) [file pcbi.1003665.s010.pdf]

SciClone: Inferring clonal architecture and tracking the spatial and temporal patterns of tumor evolution

Christopher A. Miller[1,†],Brian S. White[1,2,†], Nathan D. Dees[1], Malachi Griffith[1,6], John S. Welch[2,3], Obi L. Griffith[1,2], Ravi Vij[2,3], Michael H. Tomasson[2,3], Timothy A. Graubert[2,3,4], Matthew J. Walter[2,3,6], Matthew J. Ellis[2,3], William Schierding[5], John F. DiPersio[2,3], Timothy J. Ley[1,2,3,6], Elaine R. Mardis[1,2,3,6], Richard K. Wilson[1,2,3,6], and Li Ding[1,2,3,6,*]

1 The Genome Institute, Washington University, St. Louis, Missouri, USA
2 Department of Internal Medicine, Division of Oncology, Washington University School of Medicine, St. Louis, Missouri, USA
3 Siteman Cancer Center, Barnes-Jewish Hospital, Washington University School of Medicine, St. Louis, Missouri, USA
4 Massachusetts General Hospital, Boston, MA
5 Liggins Institute, Auckland, New Zealand
6 Department of Genetics, Washington University, St. Louis, Missouri, USA
† These authors contributed equally to this work
∗ E-mail: lding@genome.wustl.edu

A Supplementary Discussion

Each of the beta, binomial, and Gaussian mixture models available in SciClone has advantages. The binomial is theoretically most attractive for analysis of variant allele count data, while the (multivariate) Gaussian distribution can capture correlation between samples. However, we have found the beta mixture model to work best in practice. It is attractive in describing a non-negative domain, unlike the Gaussian distribution, while offering more degrees of freedom than the binomial distribution. The latter concern could be addressed by a beta-binomial (compound) distribution. However, like the binomial distribution, the beta-binomial distribution can not describe non-count-based events (e.g., cellular fractions harboring a CNA). Further, the beta-binomial distribution has no conjugate prior distribution and, hence, is not immediately amenable to the variational Bayesian approach. A similar situation occurs with the beta mixture model and was overcome by Ma and Leijon [1] through a non-linear approximation to an expectation value that yielded an approximate posterior distribution. Though a similar approach may be suitable for the beta-binomial distribution, we have not found it warranted at this time.

B Integration of copy number information

In Fig. S1, we show how copy number information can be used in our framework as an additional signal to help identify subclonal populations. In this example, we used THetA [2] to ascertain clonal and subclonal copy number events in the multiple myeloma sample. THetA estimates both the percentage of tumor cells that contain the copy number-altered regions in this sample and the normal admixture. This allowed us to calculate the VAF that would be observed for single nucleotide variants in these regions. We then added these VAFs to the SNV list and clustered as described in the main text. Fig. S1 shows the copy number-derived points highlighted in yellow (at an arbitrary coverage). Due to computational constraints, we applied THetA to a limited number of representative regions containing copy number neutral, triploid, and subclonally deleted regions.

C Bayesian variational inference

We briefly review the general approach to Bayesian variational inference, in which a probabilistic model describes the joint distribution $p(X, \mathbf{\Phi})$ and we wish to infer approximations $q(\mathbf{\Phi})$ of the posterior distribution $p(\mathbf{\Phi}|X)$. The interested reader is referred to the wealth of prior literature [3–7] providing the motivation for and derivation of this theory. In this section, $\mathbf{\Phi}$ is the set of all latent variables and parameters, including $\mathcal{Z}$ and $\boldsymbol{\pi}$, which were specified explicitly and independently of $\mathbf{\Phi}$ in Materials and Methods. The model evidence $p(X)$ may be decomposed as

$$\ln p(X) = \mathcal{L}(q) + D_{\mathrm{KL}}(q||p)$$

using the Kullback-Leibler (KL) divergence

$$D_{\mathrm{KL}}(q||p) \equiv \int q(\mathbf{\Phi}) \ln \left[\frac{q(\mathbf{\Phi})}{p(\mathbf{\Phi}|X)} \right] d\mathbf{\Phi}$$

and

$$\mathcal{L}(q) \equiv \int q(\mathbf{\Phi}) \ln \left[\frac{p(X, \mathbf{\Phi})}{q(\mathbf{\Phi})} \right] d\mathbf{\Phi} \ , \tag{S1}$$

and where any integrations over discrete variables should be interpreted as summations. This may be verified by substituting the product rule of probability $\ln p(X, \mathbf{\Phi}) = \ln p(\mathbf{\Phi}|X) + \ln p(X)$ into $\mathcal{L}(q)$ and noting that $q(\mathbf{\Phi})$ is a normalized distribution [3]. The Kullback-Leibler divergence is always non-negative, $D_{\mathrm{KL}}(q||p) \geq 0$, with equality if and only if $q(\mathbf{\Phi}) = p(\mathbf{\Phi}|X)$. Thus, $\mathcal{L}(q) \leq \ln p(X)$ and is referred to as the lower bound. Since the lower bound involves the joint distribution $p(X, \mathbf{\Phi})$, we can avoid the more difficult task of directly considering the evidence $p(X)$: our goal of finding a good approximate $q(\mathbf{\Phi})$ [i.e., with minimal Kullback-Leibler divergence from $p(\mathbf{\Phi}|X)$] is equivalent to maximizing the functional $\mathcal{L}(q)$ with respect to $q(\mathbf{\Phi})$.

To make this task tractable, we limit the domain of our maximization to a restricted family of distributions $q(\mathbf{\Phi})$. In particular, we consider factorizations

$$q(\mathbf{\Phi}) = \prod_i q_i(\Phi_i) \equiv \prod_i q_i \tag{S2}$$

that effectively partition the parameters and latent variables into independent and non-overlapping subsets Φ_i with $\mathbf{\Phi} = \cup_i \Phi_i$ and with the short-hand notation $q_i \equiv q_i(\Phi_i)$. Though this imposed independence is an approximation, no further restrictions are made on the form of the q_i, which are determined by the choice of prior distributions over $\mathbf{\Phi}$. Thus, the choice of factorization and prior are sufficient to define $q(\mathbf{\Phi})$, as we will see in the derivations of the variational Bayesian binomial and Gaussian mixture models below.

Our goal is to maximize the lower bound of Eq. (S1) with respect to the q_i, subject to the factorized form of Eq. (S2), which, substituting the latter into the form, may be written as

$$\mathcal{L}(q) = \int \prod_i q_i \left[\ln p(X, \mathbf{\Phi}) - \sum_j \ln q_j \right] d\mathbf{\Phi} \ . \tag{S3}$$

In order to perform this variational optimization with respect to each of the q_i, we begin by extracting the q_i-dependent contribution $\mathcal{L}_i(q)$ to $\mathcal{L}(q)$

$$\begin{aligned} \mathcal{L}_i(q) &\propto \int q_i \left[\int \ln p(X, \mathbf{\Phi}) \prod_{j \neq i} q_j d\Phi_j \right] d\Phi_i - \int q_i \ln q_i d\Phi_i \\ &\equiv \int q_i \ln \tilde{p}_i(X, \mathbf{\Phi}) d\Phi_i - \int q_i \ln q_i d\Phi_i \ , \end{aligned} \tag{S4}$$

where we have defined the distribution $\tilde{p}_i(X, \mathbf{\Phi})$ according to

$$\ln \tilde{p}_i(X, \mathbf{\Phi}) \equiv E_{j\neq i}\left[\ln p(X, \mathbf{\Phi})\right] + \text{const}$$

and $E_{j\neq i}[\cdot]$ denotes the expectation with respect to the distributions q_j $(j \neq i)$ comprising the approximate posterior distribution

$$E_{j\neq i}\left[\ln p(X, \mathbf{\Phi})\right] \equiv \int \ln p(X, \mathbf{\Phi}) \prod_{j\neq i} q_j d\Phi_j \ .$$

Since the q_i are normalized and independent, the cross term integrals, $\int q_i \ln q_{j\neq i} d\mathbf{\Phi} = \int \ln q_{j\neq i} d\Phi_j$, are constant with respect to q_i and do not appear in Eq. (S4). By noting that Eq. (S4) is the negative Kullback-Leibler divergence $D_{\text{KL}}(q_i||\tilde{p}_i)$, we see that the q_i^* that maximizes $\mathcal{L}_i(q)$ is defined by

$$\ln q_i^* \propto E_{j\neq i}\left[\ln p(X, \mathbf{\Phi})\right] \ . \tag{S5}$$

The constant of proportionality is fixed by the requirement that the q_i be normalized.

Eq. (S5) implicitly defines q_i^* in terms of all other q_j^* $(j \neq i)$. Therefore, to determine the q_i^*, we initialize them and then iterate, replacing each in turn with the right-hand side of Eq. (S5) evaluated using the current value of the other q_j^* $(j \neq i)$, until convergence. Convergence is guaranteed because $\mathcal{L}(q)$ is of the form of a Kullback-Leibler divergence and hence is convex with respect to each of the q_i [8].

D Variational Bayesian mixture of binomials

To directly model count data (rather than their derived ratios, the VAFs), we assume that a genomic location with x^{var} sequencing reads supporting the variant allele and x^{ref} reads supporting the reference allele and belonging to component k is described by a binomial distribution

$$\text{Bin}(x^{\text{var}}; x^{\text{ref}}, \mu_k) \equiv \binom{x^{\text{var}} + x^{\text{ref}}}{x^{\text{var}}} \mu_k^{x^{\text{var}}} (1-\mu_k)^{x^{\text{ref}}} \ .$$

As with the beta mixture model, we assume that counts are independent across samples. Hence, collecting the reads x_s^{var} and x_s^{ref} from sample s into the S-vectors $\mathbf{x}^{\text{var}} \equiv (x_1^{\text{var}}, x_2^{\text{var}}, \ldots, x_S^{\text{var}})$ and $\mathbf{x}^{\text{ref}} \equiv (x_1^{\text{ref}}, x_2^{\text{ref}}, \ldots, x_S^{\text{ref}})$, defining $\boldsymbol{\mu}_k$ as the S-vector whose s^{th} component is μ_{ks}, and instantiating the abstract notation $\boldsymbol{\chi} \equiv \mathbf{x}^{\text{var}}$ (while suppressing $\mathbf{x}^{\text{ref}}$ for notational convenience) and $\Phi_k^{\text{bin}} \equiv \boldsymbol{\mu}_k$, the analog of Eq. (1) is

$$p(\boldsymbol{\chi}|\Phi_k^{\text{bin}}) \equiv \mathbf{Bin}(\mathbf{x}^{\text{var}}; \mathbf{x}^{\text{ref}}, \boldsymbol{\mu}_k) \equiv \prod_{s=1}^{S} \text{Bin}(x_s^{\text{var}}; x_s^{\text{ref}}, \mu_{ks}) \ . \tag{S6}$$

Extending the abstract notation across all components, $\Phi^{\text{bin}} \equiv \widetilde{\boldsymbol{\mu}} \equiv \{\boldsymbol{\mu}_k\}$, we may write the analog of Eq. (3)

$$p(\boldsymbol{\chi}_n|\mathbf{z}_n, \Phi^{\text{bin}}) \equiv p(\mathbf{x}_n^{\text{var}}|\mathbf{z}_n, \widetilde{\boldsymbol{\mu}}) = \prod_{k=1}^{K} \mathbf{Bin}(\mathbf{x}_n^{\text{var}}; \mathbf{x}_n^{\text{ref}}, \boldsymbol{\mu}_k)^{z_{nk}} \ , \tag{S7}$$

describing the probability that the variant arises from the mixture. This may be combined with Eq. (2) to give the complete-data likelihood

$$p(X, \mathcal{Z}|\boldsymbol{\pi}, \Phi^{\text{bin}}) \equiv p(\mathcal{R}, \mathcal{Z}|\boldsymbol{\pi}, \widetilde{\boldsymbol{\mu}}) = \prod_{n=1}^{N} \prod_{k=1}^{K} \left[\pi_k \mathbf{Bin}(\mathbf{x}_n^{\text{var}}; \mathbf{x}_n^{\text{ref}}, \boldsymbol{\mu}_k)\right]^{z_{nk}} \ , \tag{S8}$$

where $\mathcal{R} \equiv \{\mathbf{x}_n^{\text{var}}\}$.

D.1 Prior distributions

We begin our variational Bayesian treatment of the binomial mixture model by specifying prior distributions over the parameters $\boldsymbol{\pi}$ and $\widetilde{\boldsymbol{\mu}}$. Here, convenient conjugate prior distributions exist, unlike the case of the beta mixture model. As there, we choose a Dirichlet prior distribution $\mathcal{D}(\boldsymbol{\pi}; \boldsymbol{c}^0)$ over the mixing coefficients $\boldsymbol{\pi}$, since it is conjugate to Eq. (2). Since the beta distribution is conjugate to the binomials appearing in Eq. (S7), we define the prior distribution $p(\widetilde{\boldsymbol{\mu}})$ over $\widetilde{\boldsymbol{\mu}}$ as

$$p(\widetilde{\boldsymbol{\mu}}) = \prod_{k=1}^{K} \mathbf{Beta}(\boldsymbol{\mu}_k; \mathbf{a}_k^0, \mathbf{b}_k^0) \equiv \prod_{k=1}^{K} \prod_{s=1}^{S} \mathrm{Beta}(\mu_{ks}; a_{ks}^0, b_{ks}^0) \ . \tag{S9}$$

D.2 Approximate posterior distribution

In specifying the form of the approximate posterior distribution, $q(\mathcal{Z}, \boldsymbol{\pi}, \widetilde{\boldsymbol{\mu}})$, we make the standard assumption that the latent variables and parameters factorize:

$$q(\mathcal{Z}, \boldsymbol{\pi}, \widetilde{\boldsymbol{\mu}}) = q(\mathcal{Z}) q(\boldsymbol{\pi}, \widetilde{\boldsymbol{\mu}}) \ . \tag{S10}$$

We proceed to define the concrete realizations of $q(\boldsymbol{\pi}, \widetilde{\boldsymbol{\mu}})$ and $q(\mathcal{Z})$ by applying Eq. (S5) to the joint distribution

$$p(\mathcal{R}, \mathcal{Z}, \boldsymbol{\pi}, \widetilde{\boldsymbol{\mu}}) = p(\mathcal{R}, \mathcal{Z} | \boldsymbol{\pi}, \widetilde{\boldsymbol{\mu}}) p(\boldsymbol{\pi}) p(\widetilde{\boldsymbol{\mu}}) \ , \tag{S11}$$

whose logarithm may be expressed via Eqns. (5), (S8), and (S9) as

$$\begin{aligned}
\ln p(\mathcal{R}, \mathcal{Z}, \boldsymbol{\pi}, \widetilde{\boldsymbol{\mu}}) &= \ln p(\mathcal{R}, \mathcal{Z} | \boldsymbol{\pi}, \widetilde{\boldsymbol{\mu}}) + \ln p(\boldsymbol{\pi}) + \ln p(\widetilde{\boldsymbol{\mu}}) \\
&= \sum_{n=1}^{N} \sum_{k=1}^{K} z_{nk} \left\{ \ln \pi_k + \sum_{s=1}^{S} \left[\ln \binom{x_{ns}^{\mathrm{var}} + x_{ns}^{\mathrm{ref}}}{x_{ns}^{\mathrm{var}}} + x_{ns}^{\mathrm{var}} \ln \mu_{ks} + x_{ns}^{\mathrm{ref}} \ln (1 - \mu_k) \right] \right\} \\
&\quad + \ln C(\boldsymbol{c}^0) + \sum_{k=1}^{K} \left(c_k^0 - 1 \right) \ln \pi_k \\
&\quad + \sum_{k=1}^{K} \sum_{s=1}^{S} \left\{ \ln \Gamma \left(a_{ks}^0 + b_{ks}^0 \right) - \ln \Gamma(a_{ks}^0) - \ln \Gamma(b_{ks}^0) + \left(a_{ks}^0 - 1 \right) \ln \mu_{ks} + \left(b_{ks}^0 - 1 \right) \ln (1 - \mu_{ks}) \right\} \ .
\end{aligned} \tag{S12}$$

We determine $q(\mathcal{Z})$ via Eq. (S5) as

$$\ln q(\mathcal{Z}) = \mathrm{E}_{\boldsymbol{\pi}, \widetilde{\boldsymbol{\mu}}}[\ln p(\mathcal{R}, \mathcal{Z}, \boldsymbol{\pi}, \widetilde{\boldsymbol{\mu}})] + \mathrm{const} \ .$$

Taking the expectation value of the $\mathcal{Z}$-dependent terms on the right-hand side of Eq. (S12) gives

$$\begin{aligned}
\ln q(\mathcal{Z}) &\propto \sum_{n=1}^{N} \sum_{k=1}^{K} z_{nk} \left(\mathrm{E}_{\boldsymbol{\pi}}[\ln \pi_k] + \sum_{s=1}^{S} \left\{ \ln \binom{x_{ns}^{\mathrm{var}} + x_{ns}^{\mathrm{ref}}}{x_{ns}^{\mathrm{var}}} + x_{ns}^{\mathrm{var}} \mathrm{E}_{\widetilde{\boldsymbol{\mu}}}[\ln \mu_{ks}] + x_{ns}^{\mathrm{ref}} \mathrm{E}_{\widetilde{\boldsymbol{\mu}}}[\ln (1 - \mu_k)] \right\} \right) \\
&\equiv \sum_{n=1}^{N} \sum_{k=1}^{K} z_{nk} \ln \rho_{nk} \ ,
\end{aligned}$$

with

$$\ln \rho_{nk} \equiv \mathrm{E}_{\boldsymbol{\pi}}[\ln \pi_k] + \sum_{s=1}^{S} \left\{ \ln \binom{x_{ns}^{\mathrm{var}} + x_{ns}^{\mathrm{ref}}}{x_{ns}^{\mathrm{var}}} + x_{ns}^{\mathrm{var}} \mathrm{E}_{\widetilde{\boldsymbol{\mu}}}[\ln \mu_{ks}] + x_{ns}^{\mathrm{ref}} \mathrm{E}_{\widetilde{\boldsymbol{\mu}}}[\ln (1 - \mu_k)] \right\} \ . \tag{S13}$$

Exponentiating gives

$$q(\mathcal{Z}) \propto \prod_{n=1}^{N}\prod_{k=1}^{K} \rho_{nk}^{z_{nk}} \ ,$$

with the constant of proportionality fixed by the required normalization of the distribution over 1-of-K variables as

$$q(\mathcal{Z}) = \prod_{n=1}^{N}\prod_{k=1}^{K} r_{nk}^{z_{nk}} \ ,$$

with

$$r_{nk} \equiv \frac{\rho_{nk}}{\sum_{j=1}^{K} \rho_{nj}} \ . \tag{S14}$$

Applying Eq. (S5) to determine $q(\boldsymbol{\pi}, \widetilde{\boldsymbol{\mu}})$ gives

$$\ln q(\boldsymbol{\pi}, \widetilde{\boldsymbol{\mu}}) = \mathrm{E}_{\mathcal{Z}}[\ln p(\mathcal{R}, \mathcal{Z}, \boldsymbol{\pi}, \widetilde{\boldsymbol{\mu}})] + \mathrm{const}$$

with the resulting expectation values of the $\boldsymbol{\pi}$- and $\widetilde{\boldsymbol{\mu}}$-dependent terms on the right-hand side of Eq. (S12)

$$\begin{aligned} \ln q(\boldsymbol{\pi}, \widetilde{\boldsymbol{\mu}}) \propto & \sum_{n=1}^{N}\sum_{k=1}^{K} \mathrm{E}_{\mathcal{Z}}[z_{nk}] \left\{ \ln \pi_k + \sum_{s=1}^{S} \left[x_{ns}^{\mathrm{var}} \ln \mu_{ks} + x_{ns}^{\mathrm{ref}} \ln\left(1-\mu_{ks}\right) \right] \right\} \\ & + \sum_{k=1}^{K} \left(c_k^0 - 1\right) \ln \pi_k + \sum_{k=1}^{K}\sum_{s=1}^{S} \left\{ \left(a_{ks}^0 - 1\right) \ln \mu_{ks} + \left(b_{ks}^0 - 1\right) \ln\left(1-\mu_{ks}\right) \right\} \ . \end{aligned} \tag{S15}$$

This shows that $\boldsymbol{\pi}$ and $\widetilde{\boldsymbol{\mu}}$ are decoupled, so that

$$q(\boldsymbol{\pi}, \boldsymbol{\mu}) = q(\boldsymbol{\pi}) \prod_{k=1}^{K} q(\boldsymbol{\mu}_k) \ .$$

Collecting the $\boldsymbol{\pi}$-dependent terms accordingly gives

$$\begin{aligned} \ln q(\boldsymbol{\pi}) &\propto \sum_{k=1}^{K} \left\{ \left(c_k^0 - 1\right) + \sum_{n=1}^{N} \mathrm{E}_{\mathcal{Z}}[z_{nk}] \right\} \ln \pi_k \\ &\equiv \sum_{k=1}^{K} \left(c_k - 1\right) \ln \pi_k \ , \end{aligned}$$

where

$$\begin{aligned} c_k &\equiv c_k^0 + N_k \\ N_k &\equiv \sum_{n=1}^{N} \mathrm{E}_{\mathcal{Z}}[z_{nk}] \ . \end{aligned} \tag{S16}$$

Thus, as expected from the choice of a conjugate prior, $q(\boldsymbol{\pi})$ has the form of a Dirichlet distribution, from which we can infer its normalization:

$$q(\boldsymbol{\pi}) = \mathcal{D}(\boldsymbol{\pi}; \boldsymbol{c}) \ .$$

Focusing in turn on the $\widetilde{\boldsymbol{\mu}}$-dependent terms of Eq. (S15) gives

$$\begin{aligned}\ln q(\mu_{ks}) &\propto \left\{\left(a^0_{ks}-1\right)+\sum_{n=1}^{N}\mathrm{E}_{\mathcal{Z}}[z_{nk}]x^{\mathrm{var}}_{ns}\right\}\ln\mu_{ks}+\left\{\left(b^0_{ks}-1\right)+\sum_{n=1}^{N}\mathrm{E}_{\mathcal{Z}}[z_{nk}]x^{\mathrm{ref}}_{ns}\right\}\ln\left(1-\mu_{ks}\right)\\ &\equiv a_{ks}\ln\mu_{ks}+b_{ks}\ln\left(1-\mu_{ks}\right)\ ,\end{aligned}$$

where

$$a_{ks}\equiv\left(a^0_{ks}-1\right)+N_k\bar{x}^{\mathrm{var}}_{ks} \tag{S17}$$

$$b_{ks}\equiv\left(b^0_{ks}-1\right)+N_k\bar{x}^{\mathrm{ref}}_{ks} \tag{S18}$$

$$\bar{x}^{\mathrm{var}}_{ks}\equiv\frac{1}{N_k}\sum_{n=1}^{N}\mathrm{E}_{\mathcal{Z}}[z_{nk}]x^{\mathrm{var}}_{ns}$$

$$\bar{x}^{\mathrm{ref}}_{ks}\equiv\frac{1}{N_k}\sum_{n=1}^{N}\mathrm{E}_{\mathcal{Z}}[z_{nk}]x^{\mathrm{ref}}_{ns}\ .$$

Again, as anticipated from our choice of conjugate prior, $\ln q(\mu_{ks})$ has the form of a beta distribution, from which we may infer its normalization constant:

$$q(\mu_{ks})=\mathrm{Beta}\left(\mu_{ks};a_{ks},b_{ks}\right)\ . \tag{S19}$$

Abstractly,

$$q(\Phi^{\mathrm{bin}})\equiv\prod_{k=1}^{K}\prod_{s=1}^{S}q(\mu_{ks})=\prod_{k=1}^{K}\prod_{s=1}^{S}\mathrm{Beta}(\mu_{ks};a_{ks},b_{ks})\ . \tag{S20}$$

Having determined the form and parameterization of the approximate posterior distribution, expectation values in the above equations may be evaluated using standard properties of the corresponding distributions [3]

$$\mathrm{E}_{\mathcal{Z}}[z_{nk}]=r_{nk} \tag{S21a}$$

$$\mathrm{E}_{\boldsymbol{\pi}}[\ln\pi_k]=\psi\left(c_k\right)-\psi\left(\sum_{j=1}^{K}c_j\right) \tag{S21b}$$

$$\mathrm{E}_{\widetilde{\boldsymbol{\mu}}}[\ln\mu_{ks}]=\psi\left(a_{ks}\right)-\psi\left(a_{ks}+b_{ks}\right) \tag{S21c}$$

$$\mathrm{E}_{\widetilde{\boldsymbol{\mu}}}[\ln\left(1-\mu_{ks}\right)]=\psi\left(b_{ks}\right)-\psi\left(a_{ks}+b_{ks}\right)\ , \tag{S21d}$$

where the latter two expectation values over the beta distribution are special cases of the preceding expectation value over the Dirichlet distribution and where $\psi(\cdot)$ is the digamma function

$$\psi(a)\equiv\frac{d}{da}\ln\Gamma(a)\ .$$

D.3 Variational lower bound

The variational lower bound may be calculated by substituting Eqns. (S10) and (S11) into Eq. (S1) as

$$\begin{aligned}\mathcal{L}(q)&=\sum_{\mathcal{Z}}\int q(\mathcal{Z},\boldsymbol{\pi},\widetilde{\boldsymbol{\mu}})\ln\left\{\frac{p(\mathcal{R},\mathcal{Z},\boldsymbol{\pi},\widetilde{\boldsymbol{\mu}})}{q(\mathcal{Z},\boldsymbol{\pi},\widetilde{\boldsymbol{\mu}})}\right\}d\boldsymbol{\pi}d\widetilde{\boldsymbol{\mu}}\\ &=\mathrm{E}_{\mathcal{Z},\boldsymbol{\pi},\widetilde{\boldsymbol{\mu}}}[\ln p(\mathcal{R},\mathcal{Z},\boldsymbol{\pi},\widetilde{\boldsymbol{\mu}})]-\mathrm{E}_{\mathcal{Z},\boldsymbol{\pi},\widetilde{\boldsymbol{\mu}}}[\ln q(\mathcal{Z},\boldsymbol{\pi},\widetilde{\boldsymbol{\mu}})]\\ &=\mathrm{E}_{\mathcal{Z},\widetilde{\boldsymbol{\mu}}}[\ln p(\mathcal{R}|\mathcal{Z},\widetilde{\boldsymbol{\mu}})]+\mathrm{E}_{\mathcal{Z},\boldsymbol{\pi}}[\ln p(\mathcal{Z}|\boldsymbol{\pi})]+\mathrm{E}_{\boldsymbol{\pi}}[\ln p(\boldsymbol{\pi})]+\mathrm{E}_{\widetilde{\boldsymbol{\mu}}}[\ln p(\widetilde{\boldsymbol{\mu}})]\\ &\qquad-\mathrm{E}_{\mathcal{Z}}[\ln q(\mathcal{Z})]-\mathrm{E}_{\boldsymbol{\pi}}[\ln q(\boldsymbol{\pi})]-\mathrm{E}_{\widetilde{\boldsymbol{\mu}}}[\ln q(\widetilde{\boldsymbol{\mu}})]\end{aligned} \tag{S22}$$

with

$$
\begin{aligned}
\mathrm{E}_{\mathcal{Z},\widetilde{\boldsymbol{\mu}}}[\ln p(\mathcal{R}|\mathcal{Z},\widetilde{\boldsymbol{\mu}})] &= \sum_{n=1}^{N}\sum_{k=1}^{K}\mathrm{E}_{\mathcal{Z}}[z_{nk}]\left\{\sum_{s=1}^{S}\ln\binom{x_{ns}^{\mathrm{var}}+x_{ns}^{\mathrm{ref}}}{x_{ns}^{\mathrm{var}}}+x_{ns}^{\mathrm{var}}\mathrm{E}_{\widetilde{\boldsymbol{\mu}}}[\ln\mu_{ks}]+x_{ns}^{\mathrm{ref}}\mathrm{E}_{\widetilde{\boldsymbol{\mu}}}[\ln(1-\mu_{ks})]\right\}\\
\mathrm{E}_{\mathcal{Z},\boldsymbol{\pi}}[\ln p(\mathcal{Z}|\boldsymbol{\pi})] &= \sum_{n=1}^{N}\sum_{k=1}^{K}\mathrm{E}_{\mathcal{Z}}[z_{nk}]\mathrm{E}_{\boldsymbol{\pi}}[\ln\pi_k]\\
\mathrm{E}_{\boldsymbol{\pi}}[\ln p(\boldsymbol{\pi})] &= \ln C(\boldsymbol{c}^0)+\sum_{k=1}^{K}\left(c_k^0-1\right)\mathrm{E}_{\boldsymbol{\pi}}[\ln\pi_k]\\
\mathrm{E}_{\widetilde{\boldsymbol{\mu}}}[\ln p(\widetilde{\boldsymbol{\mu}})] &= \sum_{k=1}^{K}\sum_{s=1}^{S}\left\{\ln\Gamma\left(a_{ks}^0+b_{ks}^0\right)-\ln\Gamma\left(a_{ks}^0\right)-\ln\Gamma\left(b_{ks}^0\right)\right.\\
&\qquad\left.+\left(a_{ks}^0-1\right)\mathrm{E}_{\widetilde{\boldsymbol{\mu}}}[\ln\mu_{ks}]+\left(b_{ks}^0-1\right)\mathrm{E}_{\widetilde{\boldsymbol{\mu}}}[\ln(1-\mu_{ks})]\right\}\\
\mathrm{E}_{\mathcal{Z}}[\ln q(\mathcal{Z})] &= \sum_{n=1}^{N}\sum_{k=1}^{K}\mathrm{E}_{\mathcal{Z}}[z_{nk}]\ln r_{nk}\\
\mathrm{E}_{\boldsymbol{\pi}}[\ln q(\boldsymbol{\pi})] &= \ln C(\boldsymbol{c})+\sum_{k=1}^{K}(c_k-1)\,\mathrm{E}_{\boldsymbol{\pi}}[\ln\pi_k]\\
\mathrm{E}_{\widetilde{\boldsymbol{\mu}}}[\ln q(\widetilde{\boldsymbol{\mu}})] &= \sum_{k=1}^{K}\sum_{s=1}^{S}\left\{\ln\Gamma(a_{ks}+b_{ks})-\ln\Gamma(a_{ks})-\ln\Gamma(b_{ks})\right.\\
&\qquad\left.+(a_{ks}-1)\,\mathrm{E}_{\widetilde{\boldsymbol{\mu}}}[\ln\mu_{ks}]+(b_{ks}-1)\,\mathrm{E}_{\widetilde{\boldsymbol{\mu}}}[\ln(1-\mu_{ks})]\right\}\ .
\end{aligned}
$$

Since the lower bound is guaranteed not to decrease across iterations, we detect convergence of the optimization when the difference in the lower bound between consecutive iterations is small (less than 10^{-4}). Ensuring that it does not decrease is a check on the correctness of the implementation.

D.4 Parameter and prior distribution initialization

We assign the hyperparameters $a_{ks}^0 = b_{ks}^0 = 1\ \forall k$, which gives a flat prior distribution over the μ_{ks}. As with the beta mixture model, we choose $c_k^0 = 0.001$ for all k.

Also as in the beta mixture model case, we initialize the r_{nk} according to the hard assignments computed by k-means. Initial values of the parameters c_k, a_{ks}, and b_{ks} are then computed by the update Eqns. (S16), (S17), and (S18). We then iterate the calculation of the expectation values [Eq. (S21)] and of the responsibilities [Eqns. (S13) and (S14)] via the variational E step with parameter updates [Eqns. (S16), (S17), and (S18)] via the M step until convergence of the lower bound [Eq. (S22)].

D.5 Posterior predictive density

Substituting Eqns. (S6) and (S20) into Eq. (8) defines the posterior predictive density

$$
p(\hat{\chi}|X) \approx \sum_{k=1}^{K}\frac{c_k}{\hat{c}}\prod_{s=1}^{S}\int \mathrm{Bin}(\hat{x}_s^{\mathrm{var}};\hat{x}_s^{\mathrm{ref}},\mu_{ks})\mathrm{Beta}(\mu_{ks};a_{ks},b_{ks})d\mu_{ks}
$$

for the binomial mixture model. Recalling that the above compound distribution is a beta-binomial,

$$
\int \mathrm{Bin}(x;\eta,\mu)\mathrm{Beta}(\mu;a,b)d\mu = \binom{x+\eta}{x}\frac{\mathrm{B}(x+a,\eta+b)}{\mathrm{B}(a,b)}
$$

where $\mathrm{B}(\cdot,\cdot)$ is the beta function

$$\mathrm{B}(a,b) \equiv \frac{\Gamma(a)\Gamma(b)}{\Gamma(a+b)} ,$$

we can derive the final result

$$p(\hat{\chi}|X) \approx \sum_{k=1}^{K} \frac{c_k}{\hat{c}} \prod_{s=1}^{S} \binom{\hat{x}_s^{\mathrm{var}} + \hat{x}_s^{\mathrm{ref}}}{\hat{x}_s^{\mathrm{var}}} \frac{\mathrm{B}(\hat{x}_s^{\mathrm{var}} + a_{ks}, \hat{x}_s^{\mathrm{ref}} + b_{ks})}{\mathrm{B}(a_{ks}, b_{ks})} .$$

D.6 Error bars on cluster VAFs

Statistics and Bayesian confidence intervals on cluster centers may be calculated directly from the posterior distribution $q(\mu_{ks})$ defined in Eq. (S19).

E Variational Bayesian mixture of Gaussians

A Gaussian mixture model assumes that the N VAFs $\mathbf{f}_n$ from S samples are drawn from one of K multivariate Gaussian distributions, each of which is described by an S-dimensional mean vector, $\boldsymbol{\mu}_k$, and an $S \times S$-dimensional precision (i.e., inverse covariance) matrix, $\boldsymbol{\Lambda}_k$. As before, the K-dimensional latent variable $\mathbf{z}$ indicates the Gaussian component that generated VAF $\mathbf{f}$. Hence, collecting the Gaussian parameters into aggregate parameters $\widetilde{\boldsymbol{\mu}} \equiv \{\boldsymbol{\mu}_k\}$ and $\widetilde{\boldsymbol{\Lambda}} \equiv \{\boldsymbol{\Lambda}_k\}$ and instantiating the abstract notation $\boldsymbol{\chi} \equiv \mathbf{f}$, $\Phi^{\mathrm{Gauss}} \equiv \{\widetilde{\boldsymbol{\mu}}, \widetilde{\boldsymbol{\Lambda}}\}$, and $\Phi_k^{\mathrm{Gauss}} \equiv \{\boldsymbol{\mu}_k, \boldsymbol{\Lambda}_k\}$, allows us to write the analogs to Eq. (1), i.e., the conditional probability that VAF $\mathbf{f}$ arises from a particular Gaussian component k,

$$p(\boldsymbol{\chi}|\Phi_k^{\mathrm{Gauss}}) \equiv p(\mathbf{f}|\boldsymbol{\mu}_k, \boldsymbol{\Lambda}_k) = \mathcal{N}(\mathbf{f}; \boldsymbol{\mu}_k, \boldsymbol{\Lambda}_k) \tag{S23}$$

where

$$\mathcal{N}(\mathbf{x}; \boldsymbol{\mu}, \boldsymbol{\Lambda}) = (2\pi)^{-\frac{S}{2}} |\boldsymbol{\Lambda}|^{\frac{1}{2}} \exp\left[-\frac{1}{2}(\mathbf{x}-\boldsymbol{\mu})^{\mathrm{T}} \boldsymbol{\Lambda} (\mathbf{x}-\boldsymbol{\mu})\right]$$

is the multivariate Gaussian distribution and the precision matrix $\boldsymbol{\Lambda}$ is symmetric and positive definite. The analog of Eq. (3)

$$p(\boldsymbol{\chi}_n|\mathbf{z}_n, \Phi^{\mathrm{Gauss}}) \equiv p(\mathbf{f}_n|\mathbf{z}_n, \widetilde{\boldsymbol{\mu}}, \widetilde{\boldsymbol{\Lambda}}) = \prod_{k=1}^{K} \mathcal{N}(\mathbf{f}_n; \boldsymbol{\mu}_k, \boldsymbol{\Lambda}_k)^{z_{nk}} , \tag{S24}$$

may be combined with Eq. (2) to define the complete-data likelihood

$$p(X, \mathcal{Z}|\boldsymbol{\pi}, \Phi^{\mathrm{Gauss}}) \equiv p(\mathcal{F}, \mathcal{Z}|\boldsymbol{\pi}, \widetilde{\boldsymbol{\mu}}, \widetilde{\boldsymbol{\Lambda}}) = \prod_{n=1}^{N} \prod_{k=1}^{K} \left[\pi_k \mathcal{N}(\mathbf{f}_n; \boldsymbol{\mu}_k, \boldsymbol{\Lambda}_k)\right]^{z_{nk}} . \tag{S25}$$

Specification of a conjugate prior and application of the general variational Bayesian inference framework (Section C) result in an approximate posterior distribution through steps completely analogous to those for the binomial mixture model (Section D), though involving more sophisticated algebraic manipulations. In what follows, we briefly summarize those results, which are fully derived in Ref. 3.

E.1 Prior distributions

As in both the beta and binomial mixture models, we choose the Dirichlet conjugate prior distribution of Eq. (5) over the mixing coefficients $\boldsymbol{\pi}$. The remaining parameters are also governed by conjugate prior distributions

$$p(\widetilde{\boldsymbol{\mu}}, \widetilde{\boldsymbol{\Lambda}}) = p(\widetilde{\boldsymbol{\mu}}|\widetilde{\boldsymbol{\Lambda}})p(\widetilde{\boldsymbol{\Lambda}})$$

$$p(\widetilde{\boldsymbol{\mu}}|\widetilde{\boldsymbol{\Lambda}}) = \prod_{k=1}^{K} \mathcal{N}(\boldsymbol{\mu}_k; \mathbf{m}_k^0, \beta_k^0 \boldsymbol{\Lambda}_k) \tag{S26a}$$

$$p(\widetilde{\boldsymbol{\Lambda}}) = \prod_{k=1}^{K} \mathcal{W}(\boldsymbol{\Lambda}_k; \mathbf{W}_k^0, \nu_k^0) \ , \tag{S26b}$$

where

$$\mathcal{W}(\boldsymbol{\Lambda}|\mathbf{W}, \nu) = B(\mathbf{W}, \nu)|\boldsymbol{\Lambda}|^{\frac{\nu - S - 1}{2}} \exp\left[-\frac{1}{2} \operatorname{tr}\left(\mathbf{W}^{-1}\boldsymbol{\Lambda}\right)\right]$$

is the Wishart distribution,

$$B(\mathbf{W}, \nu) \equiv |\mathbf{W}|^{-\frac{\nu}{2}} \left[2^{\frac{\nu S}{2}} \pi^{\frac{S(S-1)}{4}} \prod_{s=1}^{S} \Gamma\left(\frac{\nu + 1 - s}{2}\right)\right]^{-1} \ ,$$

and the $S \times S$ matrices $\boldsymbol{\Lambda}$ and $\mathbf{W}$ are symmetric and positive definite. The current notation differs slightly from and trivially generalizes Ref. 3, which chooses the hyperparameters to be identical across clusters, i.e., $c_k^0 = \boldsymbol{\alpha}_0$, $\mathbf{m}_k^0 = \mathbf{m}_0$, $\beta_k^0 = \beta_0 = \mathbf{0}$, $\mathbf{W}_k^0 = \mathbf{W}_0$, and $\nu_k^0 = \nu_0 \ \forall k$.

E.2 Approximate posterior distribution

As before, we make the assumption that the latent variables and parameters factorize. This and the form of the prior distributions are sufficient to induce the further factorizations

$$q(\mathcal{Z}, \boldsymbol{\pi}, \widetilde{\boldsymbol{\mu}}, \widetilde{\boldsymbol{\Lambda}}) \equiv q(\mathcal{Z})q(\boldsymbol{\pi}, \widetilde{\boldsymbol{\mu}}, \widetilde{\boldsymbol{\Lambda}}) = q(\mathcal{Z})q(\boldsymbol{\pi})q(\widetilde{\boldsymbol{\mu}}, \widetilde{\boldsymbol{\Lambda}}) = q(\mathcal{Z})q(\boldsymbol{\pi}) \prod_{k=1}^{K} q(\boldsymbol{\mu}_k, \boldsymbol{\Lambda}_k) \ , \tag{S27}$$

involving the distributions

$$q(\boldsymbol{\pi}) = \mathcal{D}(\boldsymbol{\pi}; \boldsymbol{c})$$

$$q(\boldsymbol{\mu}_k, \boldsymbol{\Lambda}_k) = q(\boldsymbol{\mu}_k|\boldsymbol{\Lambda}_k)q(\boldsymbol{\Lambda}_k)$$

$$q(\boldsymbol{\mu}_k|\boldsymbol{\Lambda}_k) = \mathcal{N}(\boldsymbol{\mu}_k; \mathbf{m}_k, \beta_k \boldsymbol{\Lambda}_k)$$

$$q(\boldsymbol{\Lambda}_k) = \mathcal{W}(\boldsymbol{\Lambda}_k; \mathbf{W}_k, \nu_k) \ .$$

These result from applying Eq. (S5) to the joint distribution

$$p(\mathcal{F}, \mathcal{Z}, \boldsymbol{\pi}, \widetilde{\boldsymbol{\mu}}, \widetilde{\boldsymbol{\Lambda}}) = p(\mathcal{F}|\mathcal{Z}, \widetilde{\boldsymbol{\mu}}, \widetilde{\boldsymbol{\Lambda}})p(\mathcal{Z}|\boldsymbol{\pi})p(\boldsymbol{\pi})p(\widetilde{\boldsymbol{\mu}}, \widetilde{\boldsymbol{\Lambda}}) \ . \tag{S28}$$

Abstractly, we have

$$q(\Phi^{\text{Gauss}}) \equiv q(\widetilde{\boldsymbol{\mu}}, \widetilde{\boldsymbol{\Lambda}}) = q(\widetilde{\boldsymbol{\mu}}|\widetilde{\boldsymbol{\Lambda}})q(\widetilde{\boldsymbol{\Lambda}}) = \prod_{k=1}^{K} \mathcal{N}(\boldsymbol{\mu}_k; \mathbf{m}_k, \beta_k \boldsymbol{\Lambda}_k) \mathcal{W}(\boldsymbol{\Lambda}_k; \mathbf{W}_k, \nu_k) \ . \tag{S29}$$

The values of the parameters $\boldsymbol{c}$, β_k, $\mathbf{m}_k$, $\mathbf{W}_k$, and ν_k are provided by the update Eqns. 10.58 and 10.60-10.63, respectively, in Ref. 3, subject to the change in naming convention between this work and that mentioned previously. As usual, the r_{nk} are given by Eq. (S14), though with $\ln \rho_{nk}$ defined as

$$\ln \rho_{nk} = \mathrm{E}_{\boldsymbol{\pi}}\left[\ln \pi_k\right] + \frac{1}{2}\mathrm{E}_{\widetilde{\boldsymbol{\Lambda}}}\left[\ln |\boldsymbol{\Lambda}_k|\right] - \frac{S}{2}\ln(2\pi) - \frac{1}{2}\mathrm{E}_{\widetilde{\boldsymbol{\mu}},\widetilde{\boldsymbol{\Lambda}}}\left[(\mathbf{f}_n - \boldsymbol{\mu}_k)^{\mathrm{T}} \boldsymbol{\Lambda}_k \left(\mathbf{f}_n - \boldsymbol{\mu}_k\right)\right] .$$

The required expectation values are provided in Eqns. 10.64-10.66 of Ref. 3.

E.3 Variational lower bound

The variational lower bound may be calculated by substituting Eqns. (S27) and (S28) into Eq. (S1) as

$$\begin{aligned}\mathcal{L}(q) &= \sum_{\mathcal{Z}} \int q(\mathcal{Z}, \boldsymbol{\pi}, \widetilde{\boldsymbol{\mu}}, \widetilde{\boldsymbol{\Lambda}}) \ln \left\{ \frac{p(\mathcal{F}, \mathcal{Z}, \boldsymbol{\pi}, \widetilde{\boldsymbol{\mu}}, \widetilde{\boldsymbol{\Lambda}})}{q(\mathcal{Z}, \boldsymbol{\pi}, \widetilde{\boldsymbol{\mu}}, \widetilde{\boldsymbol{\Lambda}})} \right\} d\boldsymbol{\pi} d\widetilde{\boldsymbol{\mu}} d\widetilde{\boldsymbol{\Lambda}} \\ &= \mathrm{E}_{\mathcal{Z},\widetilde{\boldsymbol{\mu}},\widetilde{\boldsymbol{\Lambda}}}[\ln p(\mathcal{F}|\mathcal{Z}, \widetilde{\boldsymbol{\mu}}, \widetilde{\boldsymbol{\Lambda}})] + \mathrm{E}_{\mathcal{Z},\boldsymbol{\pi}}[\ln p(\mathcal{Z}|\boldsymbol{\pi})] + \mathrm{E}_{\boldsymbol{\pi}}[\ln p(\boldsymbol{\pi})] + \mathrm{E}_{\widetilde{\boldsymbol{\mu}},\widetilde{\boldsymbol{\Lambda}}}[\ln p(\widetilde{\boldsymbol{\mu}}, \widetilde{\boldsymbol{\Lambda}})] \\ &\quad - \mathrm{E}_{\mathcal{Z}}[\ln q(\mathcal{Z})] - \mathrm{E}_{\boldsymbol{\pi}}[\ln q(\boldsymbol{\pi})] - \mathrm{E}_{\widetilde{\boldsymbol{\mu}},\widetilde{\boldsymbol{\Lambda}}}[\ln q(\widetilde{\boldsymbol{\mu}}, \widetilde{\boldsymbol{\Lambda}})] ,\end{aligned}$$

where the above expectation values are provided in Eqns. 10.71-10.77 of Ref. 3.

E.4 Parameter and prior distribution initialization

As with the beta and binomial mixture models, we choose $c_k^0 = 0.001$ for all k. To avoid biasing any particular cluster center, we initialize $\mathbf{m}_k^0 = 0$. The remaining hyperparameters, ν_k^0, $\mathbf{W}_k^0$, and β_k^0 are chosen so that (1) the prior distribution over the precision $\boldsymbol{\Lambda}_k$ is broad, (2) the "effective" precision, $\beta_k^0 \mathrm{E}[\boldsymbol{\Lambda}_k]$, governing the $\mathbf{m}_k^0$ in the Gaussian distribution gives a characteristic standard deviation over the $\mathbf{m}_k^0$, and (3) the β_k^0 are near one so that the precision $\boldsymbol{\Lambda}_k$ is closely coupled to the effective precision, $\beta_k^0 \mathrm{E}[\boldsymbol{\Lambda}_k]$, and so are constrained to intermediate values that will be optimal for the distribution over $\mathbf{m}_k^0$. To ensure (1), we choose $\mathbf{W}_k^0$ to diagonal with "large" (i.e., 10^4) off-diagonal elements. From experience, a typical cluster standard deviation is ~0.03, which implies an effective precision of 10^3. Therefore, to ensure (2) we set $\beta_k^0 \mathrm{E}[\boldsymbol{\Lambda}_k] = \beta_k^0 \nu_k^0 \mathbf{W}_k^0 = 10^3 \mathbf{I}$ or $\beta_k^0 = (10\nu_k^0)^{-1}$, with $\mathbf{I}$ the identity matrix. Finally, to satisfy (3) we set ν_k^0 as small as allowed by the constraint that it be greater than $S-1$, i.e., $\nu_k^0 = \max(1, S - 1 + \tilde{\delta})$ for $\tilde{\delta} \equiv 10^{-5}$.

Also as in the beta and binomial mixture model cases, we initialize the r_{nk} according to the hard assignments computed by k-means. We then initialize $\mathbf{m}_k$ to the centers returned by k-means and initialize $\boldsymbol{c}$, $\mathbf{W}_k$, ν_k, and β_k to their respective hyperparameter values.

E.5 Posterior predictive density

Substituting Eqns. (S23) and (S29) into Eq. (8) defines the posterior predictive density for the Gaussian mixture model

$$p(\hat{\chi}|X) \approx \sum_{k=1}^{K} \frac{c_k}{\hat{c}} \mathcal{S}t(\hat{\mathbf{f}}; \mathbf{m}_k, \mathbf{L}_k, \nu_k + 1 - S) , \tag{S30}$$

in terms of the S-dimensional Student t-distribution

$$\mathcal{S}t(\mathbf{f}; \boldsymbol{\mu}, \boldsymbol{\Lambda}, \nu) = \frac{\Gamma(\nu/2 + S/2)}{\Gamma(\nu/2)} \frac{|\boldsymbol{\Lambda}|^{1/2}}{(\nu\pi)^{S/2}} \left[1 + \frac{(\mathbf{f} - \boldsymbol{\mu})^{\mathrm{T}} \boldsymbol{\Lambda} \left(\mathbf{f} - \boldsymbol{\mu}\right)}{\nu}\right]^{-\nu/2 - S/2}$$

and the precision

$$\mathbf{L}_k = \frac{(\nu_k + 1 - S)\,\beta_k}{(1+\beta_k)}\mathbf{W}_k \ . \tag{S31}$$

E.6 Error bars on cluster VAFs

The standard deviation of variants in a cluster may be calculated from the posterior predictive density [Eq. (S30)], a Student t-distribution. Using the standard property that the covariance matrix of a Student t-distribution $\mathcal{St}(\mathbf{f};\boldsymbol{\mu},\boldsymbol{\Lambda},\nu)$ is $\mathrm{Cov}[\mathbf{f}] = \frac{\nu}{\nu-2}\boldsymbol{\Lambda}^{-1}$ (for $\nu > 2$), we may take the diagonal elements of $\frac{(\nu_k+1-S)}{(\nu_k-1-S)}\mathbf{L}_k^{-1}$ as the standard deviation of Eq. (S30).

The standard error of the mean may be calculated from

$$q(\boldsymbol{\mu}_k) = \int q(\boldsymbol{\mu}_k,\boldsymbol{\Lambda}_k)d\boldsymbol{\Lambda}_k = \int \mathcal{N}(\boldsymbol{\mu}_k;\mathbf{m}_k,\beta_k\boldsymbol{\Lambda}_k)\,\mathcal{W}(\boldsymbol{\Lambda}_k;\mathbf{W}_k,\nu_k)d\boldsymbol{\Lambda}_k \ .$$

This integral may be analytically evaluated [9] as a Student t-distribution,

$$q(\boldsymbol{\mu}_k) = \mathcal{St}(\boldsymbol{\mu}_k;\mathbf{m}_k,(\nu_k+1-S)\,\beta_k\mathbf{W}_k,\nu_k+1-S) \ .$$

Similar to the above, we make take the diagonal elements of the resulting covariance matrix, $\mathrm{Cov}[\boldsymbol{\mu}_k] = [\beta_k\,(\nu_k - S - 1)]^{-1}\,\mathbf{W}_k^{-1}$, as the standard error of the mean.

References

1. Ma Z, Leijon A (2011) Bayesian estimation of beta mixture models with variational inference. IEEE Trans Pattern Anal Mach Intell 33: 2160-73.

2. Oesper L, Mahmoody A, Raphael BJ (2013) Theta: inferring intra-tumor heterogeneity from high-throughput dna sequencing data. Genome Biology 14: R80.

3. Bishop CM (2006) Pattern recognition and machine learning. Information science and statistics. New York: Springer, 738 p. pp.

4. Svensén M, Bishop CM (2005) Robust bayesian mixture modelling. Trends in Neurocomputing 64: 235–252.

5. Attias H (1999) Inferring parameters and structure of latent variable models by variational bayes. In: Uncertainty in Artificial Intelligence. pp. 21–30.

6. Attias H (2000) A variational bayesian framework for graphical models. In: Neural Information Processing Systems.

7. Beal MJ (2003) Variational algorithms for approximate bayesian inference. Ph.D. thesis, University College London.

8. Boyd S, Vandenberghe L (2004) Convex Optimiziation. Cambridge University Press.

9. Gelman A, Carlin JB, Stern HS, Rubin DB (2004) Bayesian data analysis. Boca Raton, Florida: Chapman & Hall/CRC.
